# Conserved Metabolic Changes in Nondiabetic and Type 2 Diabetic Bariatric Surgery Patients: Global Metabolomic Pilot Study

**DOI:** 10.1155/2016/3467403

**Published:** 2016-01-10

**Authors:** Konrad Sarosiek, Kirk L. Pappan, Ankit V. Gandhi, Shivam Saxena, Christopher Y. Kang, Heather McMahon, Galina I. Chipitsyna, David S. Tichansky, Hwyda A. Arafat

**Affiliations:** ^1^Department of Surgery, Thomas Jefferson University, Philadelphia, PA 19107, USA; ^2^Metabolon, Inc., Research Triangle Park, Durham, NC 27713, USA; ^3^Department of Biomedical Sciences, University of New England, Biddeford, ME 04005, USA

## Abstract

The goal of this study was to provide insight into the mechanism by which bariatric surgical procedures led to weight loss and improvement or resolution of diabetes. Global biochemical profiling was used to evaluate changes occurring in nondiabetic and type 2 diabetic (T2D) patients experiencing either less extreme sleeve gastrectomy or a full gastric bypass. We were able to identify changes in metabolism that were affected by standard preoperation liquid weight loss diet as well as by bariatric surgery itself. Preoperation weight-loss diet was associated with a strong lipid metabolism signature largely related to the consumption of adipose reserves for energy production. Glucose usage shift away from glycolytic pyruvate production toward pentose phosphate pathway, via glucose-6-phosphate, appeared to be shared across all patients regardless of T2D status or bariatric surgery procedure. Our results suggested that bariatric surgery might promote antioxidant defense and insulin sensitivity through both increased heme synthesis and HO activity or expression. Changes in histidine and its metabolites following surgery might be an indication of altered gut microbiome ecology or liver function. This initial study provided broad understanding of how metabolism changed globally in morbidly obese nondiabetic and T2D patients following weight-loss surgery.

## 1. Introduction

Diabetes is a major public health concern in the United States because of its prevalence, considerable morbidity and mortality, and economic burden with total medical costs of 245 billion dollars in 2012 alone [1, 2]. In 2010, the prevalence rate of diabetes in the US was 9.3%, affecting older population (65 years or older) even more dramatically with the rate of 25.9% [1]. Diabetes is associated with serious complications, including coronary heart disease, stroke, kidney failure, neuropathy, blindness, and amputation, and was the seventh leading cause of death in 2010 [1, 2]. Type 2 diabetes (T2D) accounts for 90–95% of all diagnosed cases [1]. Obesity is a major risk factor for T2D [2, 3], and the risk of diabetes increases directly with BMI [2, 4, 5]. According to National Center for Health Statistics (NCHS) more than one-third of US adults (34.9 percent) were obese in 2011-2012 [6]. The medical care costs of obesity in the United States are staggering, totaling about $147 billion dollars in 2008 alone [7].

Weight loss is important therapeutic goal in obese patients with T2D, because even moderate weight loss (5%) improves insulin sensitivity [2, 8]. Bariatric surgery is the most effective weight-loss therapy and has considerable beneficial effects on diabetes and other obesity-related comorbidities [2, 9–11].

Weight-loss surgery by laparoscopic sleeve gastrectomy (SG) leads to a 40–65% reduction in excess weight and, amazingly, 56% of patients achieve resolution in their type 2 diabetes and 37% see improvement in their T2D symptoms [12]. Laparoscopic gastric bypass (GB) is a more intense surgery that typically results in a 60–70% loss of excess weight and is also characterized by improvement or resolution of diabetes [9, 12, 13].

The objective of this study was to provide insight into the mechanism by which gut/stomach rerouting leads to weight loss and the improvement or resolution of diabetes. In metabolomics, an individual's metabolic state is profiled by multiplexed measurement of many low-molecular-weight metabolites [14]. Over 4,000 such metabolites have been identified in human serum [15]. Two complementary approaches, targeted and nontargeted analyses, have evolved [16]. In targeted analysis discrete groups of chemically related metabolites (e.g., amino acids) are quantified in a biological sample. In contrast, nontargeted analysis is a more qualitative approach that surveys as many different metabolites as possible [14]. Using primarily targeted approaches, multiple studies have identified higher levels of branched-chain and aromatic amino acids in insulin-resistant, obese, and T2D individuals [17]. More recent studies demonstrated that higher levels of these amino acids are predictive of progression to T2D as well as future insulin resistance and hyperglycemia [14, 18–22]. Recently, Gall and colleagues [23] used nontargeted approach to identify plasma metabolites associated with development of insulin resistance and/or glucose intolerance. Two top-ranked metabolites were an organic acid, *α*-hydroxybutyrate (*α*-HB), and a lipid, 1-linoleoyl-glycerophosphocholine (L-GPC). Ferrannini et al. proposed fasting *α*-HB and L-GPC levels as new biomarkers to help predict dysglycemia and T2D [14, 24]. This nontargeted global metabolomic profiling represents new tool that allows the comprehensive survey of metabolism and metabolic networks to gain insight into phenotype and identify biomarker candidates. So far this approach was used to find a way to predict the progression to T2D as well as future insulin resistance and impaired glucose tolerance by serum analysis of insulin-resistant, obese individuals who progressed to T2D [14]. We took an opposite approach utilizing bariatric surgery tool as the most promising way to affect weight loss and to rectify T2D symptoms in morbidly obese patients. The range of metabolic changes that accompany weight reduction is not fully characterized. It is not known whether metabolic response is the same for all bariatric procedures, nor is it known whether there are any differences between nondiabetic and T2D patients.

## 2. Research Design and Methods

### 2.1. Serum Samples Collection

15 patients represented three disease-surgery groups: nondiabetic (non-T2D) receiving SG and T2D receiving either SG or GB surgery (Table 1). Blood samples were collected over the course of treatment for each patient at the following times: at baseline (BL) prior to dieting/surgery, 14 days after baseline with adherence to strict preoperation weight-loss liquid diet (preop diet), and 28 days after surgery recovery after bariatric surgery (postop). Blood samples were collected in serum separator tubes, allowed to stand at room temperature for 15–20 minutes, centrifuged at 2500 rpm for 10 minutes at 4°C, aliquoted, snap frozen in liquid nitrogen, and stored at −80°C until analysis.

### 2.2. Global Metabolomic Analysis

Nontargeted global metabolomic analysis was performed by Metabolon, Inc. (Durham, NC), using two independent platforms: ultrahigh performance liquid chromatography/tandem mass spectrometry (UHPLC-MS/MS) optimized for basic species or acidic species, and gas chromatography/mass spectrometry (GC/MS). General platform methods are described in details in Online Supplemental Data section (see Supplementary Methods and Materials available online at http://dx.doi.org/10.1155/2016/3467403).

Following log transformation and imputation with minimum observed values for each compound, repeated measures 2-way ANOVA with posttest contrasts was used to identify biochemicals that differed significantly between experimental groups and across study time points with statistical cut-offs for *P* value (*P* < 0.05). Multiple comparisons were accounted for by estimating the false discovery rate using *q*-values of less than 5% (*q* < 0.05) [25].

## 3. Results and Discussion

### 3.1. Metabolite Summary and Significantly Altered Biochemicals

The search continues to identify biomarkers capable of predicting the onset of T2D [14]. Genome-wide association studies have identified many T2D susceptibility genes [14, 26] but generally failed to improve risk prediction over that provided by routine clinical measures [14, 27]. Global nontargeted analysis performed in this study is the first study to provide the insight into mechanism by which bariatric surgery leads to weight loss and resolution or improvement of T2D. This approach might also be used to identify T2D biomarker candidates and find new, cost effective treatments that can replace surgery itself.

Since metabolomic profiling generates a wealth of data that must be parsed to extract information, we chose statistical cut-offs at both the level of individual metabolites—*P* values—and the level of multiple testing across the 476 metabolites detected in the serum samples—*q*-values. By narrowing in on metabolites meeting the conservative criteria of *P* < 0.05 and an estimated false discovery rate of less than 5% (*q* < 0.05), we were able to reduce the complexity of the dataset and observed a number of statistically significant changes that occurred in common in nondiabetic and T2D patients with SG or GB. Furthermore, we were able to identify concerted changes of related metabolites that pointed to areas of metabolism that were affected by standard preop diet as well as by bariatric surgery itself. A list of all 476 metabolites detected and heat map of the statistical comparisons across time and patient groups are presented in Online Supplemental Tables A1 and A2.

Comparison of serum profiles at baseline, following a preop weight reduction diet, and after weight-loss surgery revealed several key metabolic differences as highlighted below.

Fat mobilization and oxidation were the key signatures associated with preop diet. Prior to surgery, patients were subjected to 2-week clear liquid diet that promoted weight loss on the order of 3–5% of body weight. The preoperative liquid diet is a 14-day high protein, very low calorie diet (VLCD) designed to deplete glycogen and fat stores in the liver or “shrink the liver” which is lifted to access the stomach during surgery. This VLCD includes 800 kcal with 80 g protein and typically produces a 10–20-pound weight loss. High protein drinks with less than 200 calories and at least 20 g protein are consumed 3-4x daily; no solid food is allowed on this diet. In addition, at least 64 ounces of sugar-free decaffeinated clear liquids a day are recommended along with a multivitamin and a calcium + vitamin D supplement. Medications, such as antihyperglycemics, are adjusted during this preoperative weight-loss phase to account for decreased calorie and carbohydrate intake. The study found that patients who follow a preoperative liquid diet effectively reduced visceral fat and achieve greater weight loss [28].

**Figure 1:**
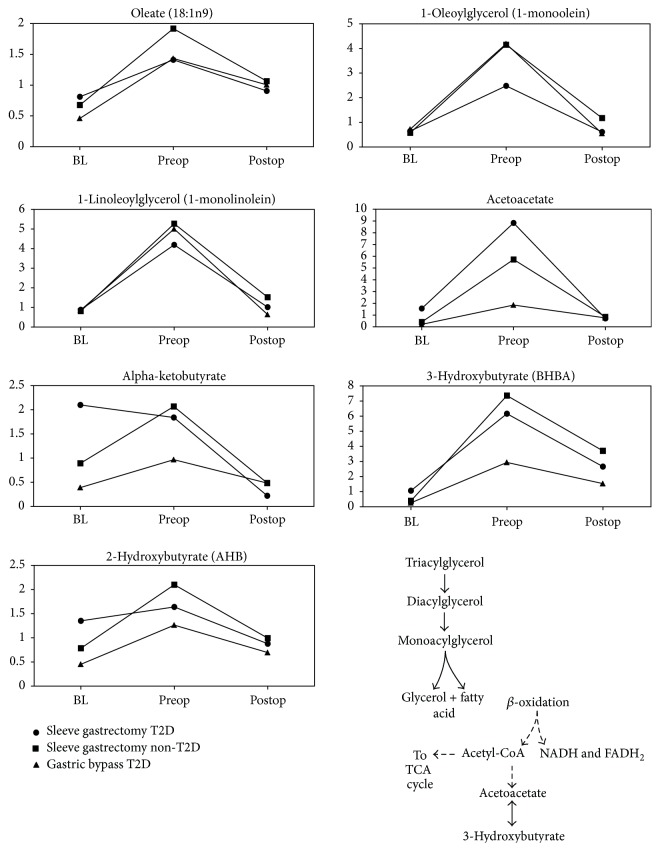
Fat mobilization and oxidation: key signatures associated with preop diet. All shown metabolites meet the conservative criteria of *P* < 0.05 and an estimated false discovery rate of less than 5% (*q* < 0.05).

Examination of preop metabolic profiles, serum samples taken immediately before surgery, showed a profound mobilization of fat as attested by statistically significant elevations of ketones, monoacylglycerols, oleate, and an acyl-carnitine (Online Supplemental Table A3, Figure 1). These are compounds associated with lipolysis and fatty acid oxidation which suggested that a major metabolic effect of the preop diet was to stimulate fat tissue triglyceride hydrolysis, transport of fatty acids to the liver, and subsequent liver fatty acid oxidation and ketogenesis to supply energy substrates for peripheral tissues. The elevation of the markers associated with lipolysis and ketone production was transient and, in most cases, returned to near baseline levels by day 28 postsurgery time point. These results suggest that 3–5% weight loss experienced by patients during preop diet is largely due to the consumption of adipose reserves for energy production.

Another interesting observation is that preop diet led to a transient elevation of alpha-hydroxybutyrate (*α*-HB) and its precursor alpha-ketobutyrate (Online Supplemental Table A3, Figure 1). *α*-HB is a sensitive biomarker of insulin resistance [23, 24] which suggests that both nondiabetic and T2D patients experienced a temporary relative increase in insulin resistance during preop diet.

**Figure 2:**
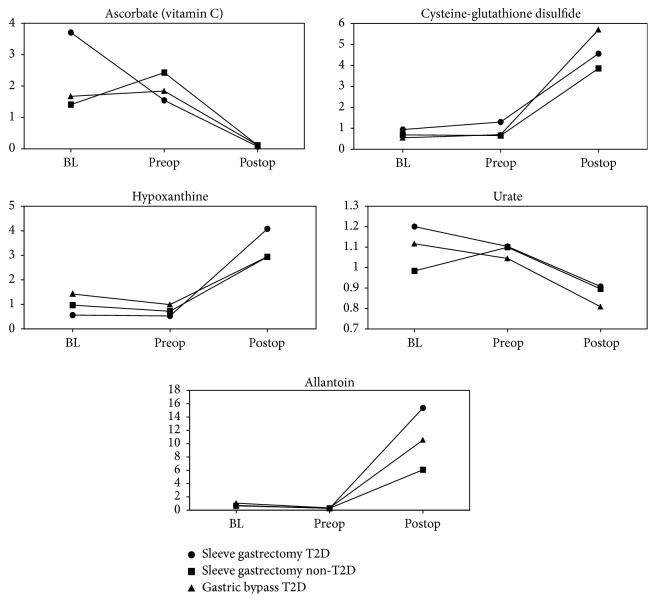
Postsurgery metabolic changes. All shown metabolites meet the conservative criteria of *P* < 0.05 and an estimated false discovery rate of less than 5% (*q* < 0.05).

Compounds that changed in a statistically significant manner after 28 days of recovery from bariatric surgery, relative to baseline, were more numerous and diverse than observed in response to the preoperation diet. 62 compounds in the postsurgery to baseline comparison represented *P* < 0.05 and showed *q* < 0.05 in at least one of the disease-surgery groups (Online Supplemental Table A4). 28 compounds showed *P* and *q*-value cut-offs across all three disease-surgery groups at the 28-day postsurgery sample collection time point relative to baseline. 13 of the compounds that changed across all three groups had fold-change increases—including 100-fold + increases for* trans*-urocanate,* cis*-urocanate, pyroglutamylvaline, and heme in most or all of the groups. The remaining fifteen compounds that changed across all three groups were reduced at the postsurgery time point compared to baseline. Levels of ascorbate and various tocopherols were substantially reduced with ascorbate showing a 12.5-fold or greater decrease in each of the groups (Online Supplemental Table A4, Figure 2). Difficulty in absorbing micronutrients, such as vitamin C, following bariatric surgery has been reported previously [29, 30] and appeared to be confirmed at a shorter follow-up time point in this study.

Glutathione and its precursors were more abundant following weight-loss surgery. Weight-loss surgery led to concerted changes in compounds related to sulfur-containing amino acid metabolism that were largely shared across the groups. Glutathione (GSH) is a tripeptide comprised of glutamate, cysteine, and glycine. These amino acids along with the recycling intermediates Cys-Gly and 5-oxoproline were increased in all groups following surgery (Online Supplemental Table A4), suggesting a greater potential availability of substrates for GSH production. Oxidized forms of glutathione and cysteine, such as the mixed heterodimer cysteine-glutathione and glutathione homodimer GSSG, were elevated following surgery (Online Supplemental Table A4, Figure 2) and could be a sign of increased oxidative stress following surgery. However, an alternate interpretation is that a greater availability of glutathione and sulfur-containing amino acids following weight-loss surgery led to the greater formation of these oxidized compounds.

**Figure 3:**
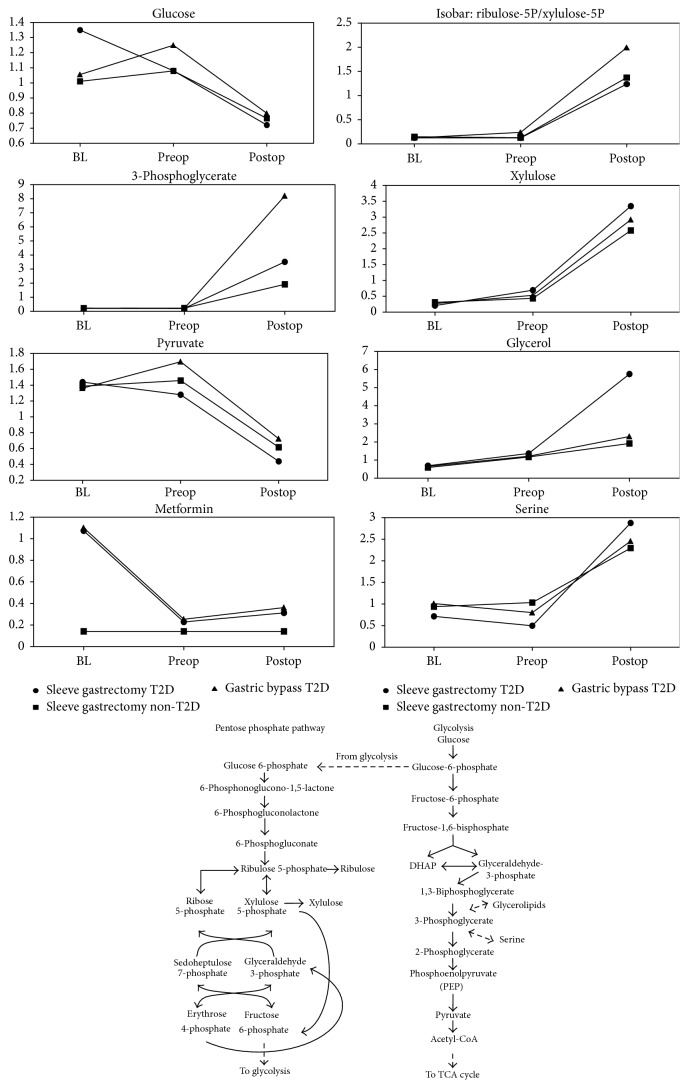
Postsurgery glucose usage shift towards pentose phosphate pathway. All shown metabolites meet the conservative criteria of *P* < 0.05 and an estimated false discovery rate of less than 5% (*q* < 0.05).

Weight-loss surgery appeared to shift glucose usage away from glycolytic pyruvate production. Pyruvate—the terminal product of glucose metabolism via the glycolysis pathway—dropped sharply after bariatric surgery (Online Supplemental Table A4, Figure 3), likely indicating its more efficient mitochondrial utilization. The reduction of pyruvate was matched by increases in fumarate, in all T2D patients, and malate perhaps indicating an inadequate supply of acetyl-CoA, which is derived from pyruvate, relative to the level of TCA cycle components. However, levels of the glycolytic intermediate 3-phosphoglycerate (3-PG) increased after surgery as did nonglycolytic products—glycerol and serine—potentially derived from 3-PG.

In addition to changes in pyruvate production, glucose usage via the pentose phosphate pathway (PPP) was also shifted following bariatric surgery. The PPP is a key source of pentose sugars used for nucleotide synthesis as well as NADPH which is used for reductive synthesis reactions and regeneration of reduced glutathione. PPP intermediates and derivative pentose sugars, including ribulose-5-phosphate and xylulose-5-phosphate that are isobars that cannot be differentiated by our platform, and their nonphosphorylated products, such as xylulose, were significantly increased in all groups following surgery (Online Supplemental Table A4, Figure 3). Glucose carbons, via glucose-6-phosphate, may have been directed toward the pentose phosphate pathway in the face of the proposed decrease in glycolysis pathway activity. Glucose metabolism was likely confounded by the use of antidiabetic medications. For example, at baseline, metformin was detected in 100% of the T2D SG patient samples, 60% of the T2D GB samples, and none of the nondiabetic SG samples. After bariatric surgery, metformin was only detected in 20% of the T2D SG and GB serum samples. Postsurgery serum glucose levels decreased relative to baseline but this change only reached statistical significance (*P* < 0.05) in the T2D SG group (Online Supplemental Table A4, Figure 3). In total, the results suggest that bariatric surgery affected glucose metabolism through glycolytic and nonglycolytic pathways similarly for all three of the disease-surgery groups.

Increased serum heme levels were a possible indication of improved liver function following surgery. Each of the patient groups experienced an increase in serum heme levels around 100-fold compared to their respective baseline levels following surgery (Online Supplemental Table A4). A couple of interesting possibilities, such as a reduced level of heme breakdown by heme oxygenase (HO) or an increased level of synthesis by 5-aminolevulinate synthase (ALA synthase), could explain these changes. The understanding of HO-1 function has evolved beyond a simple disposal of heme to include cytoprotective, anti-inflammatory, and antioxidant functions. For instance, endogenous carbon monoxide produced by HO-1 engages multiple signal transduction pathways to confer antiapoptotic and anti-inflammatory effects and biliverdin and bilirubin are potent antioxidants. HO activation has been shown to have insulin sensitizing and anti-inflammation effects in T2D [31]. So the increase in heme and biliverdin following surgery could represent an increase in heme oxidation by HO leading to greater antioxidant protection and insulin sensitivity. On the other hand, the greater availability of glycine, which shows a relative deficiency in T2D [23, 32], could also serve as the basis for greater heme production by ALA synthase—the rate-limiting enzyme of heme formation whose expression is repressed by glucose [33]. On the other hand, biliverdin catabolism—which can reflect red blood cell turnover and heme disposal—was less evident following surgery as indicated by reductions in bilirubin ZZ and its EE photoisomer (Online Supplemental Table A4). Together, these exciting results suggest that bariatric surgery may promote antioxidant defense and insulin sensitivity through both increased heme synthesis and HO activity or expression.

Diabetes and obesity are chronic conditions associated with elevated oxidative/inflammatory activities with a continuum of tissue insults leading to more severe cardiometabolic and renal complications including myocardial infarction and end-stage-renal damage [31]. A common denominator of these chronic conditions is the enhanced levels of cytokines like tumour necrosis factor-alpha (TNF-*α*), interleukin (IL-6), IL-1beta, and resistin, which in turn activates the c-Jun-N-terminal kinase (JNK) and NF-*κ*B, pathways, creating a vicious cycle that exacerbates insulin resistance, type-2 diabetes, and related complications [31]. Emerging evidence indicates that heme oxygenase (HO) inducers are endowed with potent antidiabetic and insulin sensitizing effects besides their ability to suppress immune/inflammatory response [31]. Importantly, the HO system abates inflammation through several mechanisms including the suppression of macrophage-infiltration and abrogation of oxidative/inflammatory transcription factors like NF-*κ*B, JNK, and activating protein-1 [31]. Thus, HO system could be explored in the search for novel remedies against T2D and its complications.

Ferrannini et al. proposed using fasting *α*-HB and L-GPC levels as new biomarkers to help predict dysglycemia and T2D [14, 24]. Both were detected in this study but postsurgery results do not bear out an improvement in insulin resistance based on these markers. *α*-HB is positively but L-GPC is negatively correlated with insulin resistance, so a postsurgery signature of improved insulin sensitivity would be expected to show a decrease of *α*-HB and an increase of L-GPC. Our findings showed an opposite pattern: *α*-HB was increased during the liquid weight-loss diet and then returned to near baseline levels after the surgery, while L-GPC levels showed significant postsurgery decrease across all three disease-surgery groups (Online Supplemental Table A4, Figure 1). There could be several reasons for this to occur, including the assumption that *α*-HB will drop after bariatric surgery is incorrect, or the 28-day time point is too soon to register a change. For the T2D subjects, there is the potential that metformin therapy also altered the baseline levels of *α*-HB and L-GPC. Future research is needed to clarify these findings.

**Figure 4:**
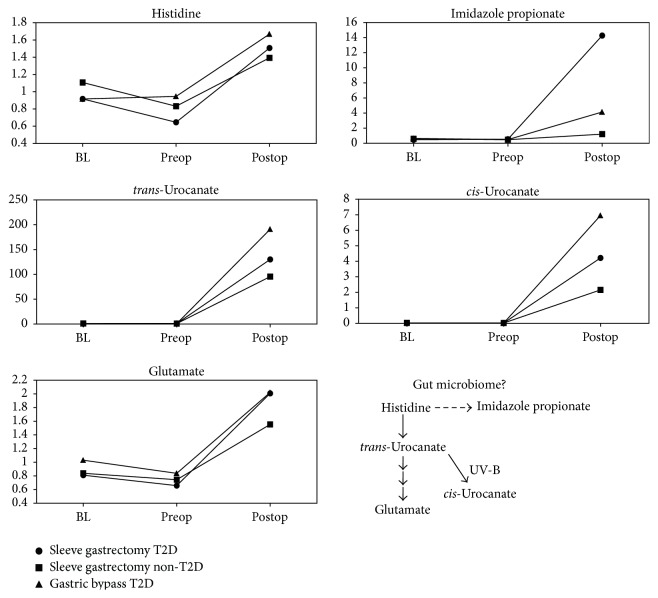
Postsurgery increase in histidine derivatives. All shown metabolites meet the conservative criteria of *P* < 0.05 and an estimated false discovery rate of less than 5% (*q* < 0.05).

Large increases in histidine derivatives were possibly due to altered gut microbiome composition or increased liver histidine-ammonia lyase activity. Histidine and several catabolites, such as imidazole propionate and urocanate isomers, both* trans*- and* cis*-urocanate, were significantly elevated (*P* < 0.05, *q* < 0.00001, including 100-fold + increases for* trans*-urocanate and* cis*-urocanate) in all three groups (Online Supplemental Table A4, Figure 4). Histidine is classified as an essential amino acid but gut bacteria can synthesize it, perhaps using precursors supplied by the human host. These markers may be an indication of changes in gut microbiome as the direct participation of the rat intestinal flora in the degradation of urocanate to imidazole propionate has been demonstrated previously [34].

Although the sample size was very small, these results suggested that histidine metabolites could also be important marker candidates to monitor metabolic changes associated with weight-loss surgery. Recently, Ryan and colleagues [35] found that vertical sleeve gastrectomy that led to weight loss and improvement of diabetes also resulted in changes in the gut bacteria. The researchers observed changes in several key bacterial groups that have been previously linked to the risk of T2D, and these changes were related to increase in circulating of bile acids that are known to bind to the nuclear receptor FXR. Interesting is the researches proposal that manipulating the gut bacteria might be another way to mimic the surgery [35].

On the other hand, urocanate is also formed in the liver by histidine-ammonia lyase (HAL) which converts histidine into urocanate and ammonia. Interestingly, HAL gene expression in hepatocytes can be stimulated by glucagon [36], so it is also possible that the increase of urocanate following surgery reflects a change in circulating glucagon levels.* trans*-Urocanate is converted to* cis*-urocanate by sunlight.* cis*-Urocanate has interesting immunosuppressive properties that are believed to help protect the skin during sun exposure and perhaps sites distal from the skin [37]. Little is known about imidazole propionate but it is a reported constituent of urine and has been proposed as a marker of intestinal dysfunction [38]. It may be useful to validate the ability of* trans*-urocanate,* cis*-urocanate, and imidazole propionate to serve as markers to monitor bariatric surgery in a larger independent cohort of patients and targeted quantitative assay. It will also be interesting to determine what, if any, utility such markers have for predicting long-term patient outcomes following surgery.

Comparing the postsurgery to the preoperation diet time point revealed 18 compounds that met the *P* and *q*-value cut-off criteria across all three disease-surgery groups (Online Supplemental Table A5). Thirteen were increased postsurgery samples relative to the samples collected at the preoperation diet time point and* trans*-urocanate,* cis*-urocanate, and pyroglutamylvaline displayed 100-fold or greater increases in nearly all of the groups.

Ascorbate and 1-linolenoylglycerol showed the greatest reductions among the 5 compounds that decreased in postsurgery samples relative to preoperation diet samples following surgery, but these reductions could also reflect altered gut absorption of these vitamins in addition to their consumption via the quenching of reactive oxygen species.

There were 29 additional compounds that represented *P* < 0.05 in all groups but did not reach *q* < 0.05 for all of the disease-surgery combinations. Histidine and several catabolites, such as imidazole propionate and urocanate isomers, were increased in T2D patients and the urocanate isomers were also likewise increased in nondiabetic patients after surgery (Online Supplemental Tables A4 and A5). Again, these results suggest that histidine metabolites could be important markers to monitor metabolic changes associated with weight-loss surgery.

## 4. Conclusions

Global metabolomic analysis was used to evaluate the changes occurring in nondiabetic and T2D patients experiencing either less extreme sleeve gastrectomy or a full gastric bypass. This study allowed gaining insights into the metabolic changes during both the preoperation weight-loss diet and early postsurgery recovery that accompany bariatric surgery.

To identify metabolic changes that were conserved across nondiabetic and T2D patients and different bariatric surgery procedures—sleeve gastrectomy (SG) versus gastric bypass (GB)—the metabolomic data collected for each disease-surgery combination were filtered according to statistical cut-offs for *P* value (*P* < 0.05) and to establish an estimated false discovery rate of less than 5% (*q* < 0.05).

It is important to point out that, despite age and sex difference, T2D status or bariatric surgery procedure, and coexistence of other associated diseases, all patients demonstrated striking similarity in major metabolome changes associated with preoperation weight-loss diet and bariatric surgery itself.

The preoperation weight-loss diet was associated with a strong lipid metabolism signature related to triglyceride hydrolysis, fatty acid oxidation, and ketone formation.

Diverse changes across a variety of metabolic areas were observed after bariatric surgery. Glucose metabolism via glycolytic and nonglycolytic pathways appeared to share a similar response across all patients regardless of baseline T2D status or the bariatric surgery procedure. Glycolysis pathway appeared to be suppressed and perhaps led to an accumulation of the TCA cycle components: malate and fumarate. Glucose derivatives in the pentose phosphate pathway were elevated following surgery. Such increases might indicate a greater demand for pentose sugars and NADPH and the redirection of glucose-6-phosphate away from glycolysis.

Increased heme levels were a likely sign of improved antioxidant defense via the action of heme oxygenase and liver function through increased heme biosynthesis in the liver. The increased availability of glutathione precursors suggested a greater capacity to synthesize glutathione. The simultaneous postsurgery disappearance of vitamin C and surge in oxidative stress markers such as allantoin and cysteine-glutathione disulfide suggest that micronutrient status should be monitored and supported by nutritional supplementation. This initial study provided a broad understanding of how metabolism changed globally in morbidly obese subjects following weight-loss surgery. Future serum metabolomic profiling studies focusing on baseline and 28 days (or other) after surgery with a greater number of patients in each group might help to further resolve differences between diabetic and nondiabetic patients. Additionally, profiling of baseline and postsurgery fecal samples might provide a more focused manner to interrogate changes associated with gut and microbiome function. Finally, the significance of this study lays in the exploration of future treatments for obesity and T2D that can mimic bariatric surgery weight loss and improvement and resolution of T2D.

## Supplementary Material

Supplementary Methods and Materials.
15 patients represented three disease-surgery groups: non-diabetic (non-T2D) receiving SG and T2D receiving either SG or GB surgery (Table1). Blood samples were collected over the course of treatment for each patient at the following times: at baseline (BL) prior to dieting/surgery, 14-days post-baseline with adherence to strict pre-operation weight-loss liquid diet (Pre-Op Diet), and 28-days post-surgery recovery after bariatric surgery (Post-Op). Blood samples were collected in serum separator tubes, allowed to stand at room temperature for 15-20 minutes, centrifuged at 2500 rpm for 10 minutes at 40°C, aliquoted, snap frozen in liquid nitrogen, and stored at -800°C until analysis.
A Global Metabolomic Study: Statistical data associated with all 476 compounds detected, p < 0.05, q < 0.05.

## Figures and Tables

**Table 1:** Clinicopathological characteristics of the patients.

Average	Gastric sleeve with T2D	Gastric sleeve without T2D	Gastric bypass with T2D
M, number of patients	2	0	1
F, number of patients	3	5	4

Age, years	46 ± 12.84	45.2 ± 14.24	44.4 ± 17.57
Weight, lb	306.28 ± 46.27	250.4 ± 18.58	304.4 ± 44.11

BMI	48.74 ± 8.2	43.54 ± 4.13	47.56 ± 6.61
Fasting blood sugar, mg/dL	140.6 ± 26.03	87 ± 2.92	98.8 ± 21.71

HTN, number of patients	2	3	3
