# Supplementary material for: Conserved Metabolic Changes in Nondiabetic and Type 2 Diabetic Bariatric Surgery Patients: Global Metabolomic Pilot Study
Source: J Diabetes Res. 2016 Jan 10;2016:3467403. doi: 10.1155/2016/3467403 (PMC4736952; doi:10.1155/2016/3467403)
Supplement: Supplementary file 1 — Supplementary Methods and Materials. 15 patients represented three disease-surgery groups: non-diabetic (non-T2D) receiving SG and T2D receiving either SG or GB surgery (Table1). Blood samples were collected over the course of treatment for each patient at the following times: at baseline (BL) prior to dieting/surgery, 14-days post-baseline with adherence to strict pre-operation weight-loss liquid diet (Pre-Op Diet), and 28-days post-surgery recovery after bariatric surgery (Post-Op). Blood samples were collected in serum separator tubes, allowed to stand at room temperature for 15-20 minutes, centrifuged at 2500 rpm for 10 minutes at 40°C, aliquoted, snap frozen in liquid nitrogen, and stored at -800°C until analysis. A Global Metabolomic Study: Statistical data associated with all 476 compounds detected, p < 0.05, q < 0.05. [file 3467403.f1.pdf]

Methods and Materials.

Non-targeted global metabolomics was performed by Metabolon, Inc. (Durham, NC) and employed two independent platforms: ultrahigh performance liquid chromatography/tandem mass spectrometry (UHPLC-MS/MS) optimized for basic or acidic species, and gas chromatography/mass spectrometry (GC/MS) (1,2).

For each serum sample, 100 µL was used for analyses.

Using an automated liquid handler (Hamilton LabStar, Salt Lake City, UT), protein was precipitated with methanol that contained four standards to report on extraction efficiency. The resulting supernatant was split into equal aliquots for analysis on two platforms. Aliquots, dried under nitrogen and vacuum-desiccated, were subsequently either reconstituted in 50 µL 0.1% formic acid in water (acidic conditions) or in 50 µL 6.5 mM ammonium bicarbonate in water, pH 8 (basic conditions) for the two UHPLC-MS/MS analyses or derivatized to a final volume of 50 µL for GC/MS analysis using equal parts bistrimethyl-silyl-trifluoroacetamide and solvent mixture acetonitrile: dichloromethane: cyclohexane (5:4:1) with 5% triethylamine at 60°C for 1 hour. In addition, three types of controls were analyzed in concert with the experimental samples: aliquots of a well-characterized human plasma pool served as technical replicates throughout the data set, extracted water samples served as process blanks, and a cocktail of standards spiked into every analyzed sample allowed instrument performance monitoring. Experimental samples and controls were randomized across platform run days.

UHLC-MS/MS samples were separated using a Waters Acquity UPLC (Waters, Millford, MA) instrument with separate acid/base-dedicated 2.1 mm × 100 mm Waters BEH C18 1.7 µm particle columns heated to 40°C and analyzed using an LTQ mass spectrometer (Thermo Fisher Scientific, Inc., Waltham, MA) which consisted of an electrospray ionization (ESI) source and linear ion-trap (LIT) mass analyzer (1). Extracts reconstituted in formic acid were gradient eluted at 350 µL/min using (A) 0.1% formic acid in water and (B) 0.1% formic acid in methanol (0% B to 70% B in 4 min, 70-98% B in 0.5 min, 98% B for 0.9 min), whereas extracts reconstituted in ammonium bicarbonate used (A) 6.5 mM ammonium bicarbonate in water, pH 8, and (B) 6.5 mM ammonium bicarbonate in 95/5 methanol/water (same gradient profile as above) at 350 µL/min. The MS instrument scanned 99-1000 m/z and alternated between MS and MS2 scans

using dynamic exclusion with approximately 6 scans per second. Derivatized samples for GC/MS were separated on a 5% diphenyl/ 95% dimethyl polysiloxane fused silica column with helium as the carrier gas and a temperature ramp from 60°C to 340°C and then analyzed on a Thermo-Finnigan Trace DSQ MS (Thermo Fisher Scientific, Inc.) operated at unit mass resolving power with electron impact ionization and a 50-750 atomic mass unit scan range (2).

Metabolites were identified by automated comparison of the ion features in the experimental samples to a reference library of chemical standard entries that included retention time, molecular weight (m/z), preferred adducts, and in-source fragments as well as associated MS spectra, and were curated by visual inspection for quality control using software (3). For statistical analyses and data display purposes, any missing values were assumed to be below the limits of detection and these values were imputed with the compound minimum. Statistical analysis of log-transformed data was performed using "R" (http://cran.r-project.org/), which is a freely available, open-source software package. Repeated measures 2-way ANOVA with post-test contrasts was performed to compare data between experimental groups and across study time points. Multiple comparisons were accounted for by estimating the false discovery rate using q-values (4).

References.

1. Evans, A.M., DeHaven, C.D., Barrett, T., Mitchell, M. and Milgram, E. Integrated, nontargeted ultrahigh performance liquid chromatography/electrospray ionization tandem mass spectrometry platform for the identification and relative quantification of the small-molecule complement of biological systems. Anal. Chem. 2009; 81:6656-6667.

2. Bourdonck, K.J., Mitchell, M.W., Nemet, L., Keresztes, L., Nyska, A., Shinar, D., Rosenstock, M. Discovery of metabolomics biomarkers for early detection of nephrotoxicity. Toxicol. Pathol. 2009; 37:280-292.

3. DeHaven C.D., Evans A.M., Dai H., Lawton K.A. Organization of GC/MS and LC/MS Metabolomics data into Chemical Libraries. J. Cheminform 2010; 2(1):9.

4. Storey J.D., Tibshirani R. Statistical significance for genomewide studies. Proc. Natl. Acad. Sci. USA 2003; 100:9440-9445.

Table A1. A Global Metabolomic Study: Statistical data associated with all 476 compounds detected.

* Green and red shaded cells indicate p≤0.05 (green indicates that the mean values are significantly higher for that comparison; red - values significantly lower). Light green and light red shaded cells indicate 0.05<p<0.10 (light green indicates that the mean values trend higher for that comparison; light red - values trend lower.

Biochemical Name	Heat map of statistically significant biochemicals*								
	Fold Change and ANOVA Contrasts								
	Pre-Op to BL Comparison			Post-Op to BL Comparison			Post-Op to Pre-Op Comparison		
	SG Non-T2D	SG T2D	GB T2D	SG Non-T2D	SG T2D	GB T2D	SG Non-T2D	SG T2D	GB T2D
glycine	1	0.78	**0.64**	1.57	**2.19**	1.16	1.57	**2.83**	**1.81**
N-acetylglycine	1.8	**4.79**	1.44	1.64	1.55	1.83	0.91	0.32	1.27
dimethylglycine	0.73	0.84	0.96	**0.57**	**0.61**	0.71	0.78	0.73	0.74
betaine	**0.76**	0.82	**0.77**	0.96	0.97	0.94	**1.25**	1.17	**1.22**
serine	1.1	0.7	0.79	**2.44**	**4.03**	**2.43**	**2.22**	**5.79**	**3.07**
N-acetylserine	1	0.82	1.08	**4.09**	**6.57**	**8.6**	**4.1**	**7.97**	**8**
beta-hydroxypyruvate	1.17	1.02	1.13	0.93	0.69	0.65	0.79	0.67	**0.57**
threonine	0.86	0.73	0.8	1.34	1.45	1.65	**1.57**	**1.99**	**2.05**
N-acetylthreonine	0.87	0.72	1.29	1.3	1.1	1.59	1.49	**1.54**	1.23
homoserine	1.4	**0.42**	0.91	0.62	0.59	0.48	0.44	1.42	0.52
alanine	0.69	**0.47**	0.71	0.63	0.8	**0.52**	0.91	**1.7**	0.73
N-acetylalanine	1.03	0.85	0.94	0.94	1.07	1.01	0.91	1.26	1.07
aspartate	0.91	0.72	0.67	**2.01**	**3.58**	1.37	**2.22**	**4.94**	**2.03**
asparagine	0.92	0.94	**0.53**	0.92	**1.58**	0.72	1.01	**1.68**	1.35
glutamate	0.89	0.81	0.81	**1.85**	**2.48**	**1.96**	**2.09**	**3.05**	**2.41**
glutamine	0.88	0.81	**0.78**	0.88	**0.75**	0.91	1.01	0.93	1.17
pyroglutamine*	1.16	0.82	0.98	**2.17**	**1.71**	**1.34**	**1.87**	**2.09**	1.36
histidine	0.75	**0.7**	1.03	1.26	**1.64**	**1.82**	**1.68**	**2.34**	**1.76**
1-methylhistidine	0.81	0.73	1.02	1.4	2.08	3.91	1.73	2.87	3.83
3-methylhistidine	2.26	6.83	0.45	0.78	1.66	0.38	0.34	0.24	0.84
trans-urocanate	0.92	1.83	1.33	**103.87**	**268.09**	**353.71**	**113.05**	**146.41**	**266.35**
cis-urocanate	0.77	1.44	1.26	**76.94**	**329.32**	**543.33**	**99.74**	**229.09**	**431.96**
imidazole propionate	0.8	1.14	0.76	2.07	**30.7**	**6.51**	2.59	**27.03**	**8.56**
lysine	0.91	0.76	0.71	**1.43**	1.15	1.27	**1.57**	**1.52**	**1.79**
glutarylcarnitine (C5)	0.89	0.75	0.96	0.83	**0.67**	**0.79**	0.92	0.89	0.83
pipecolate	1.26	0.87	**0.49**	0.9	0.79	**0.63**	0.72	0.91	1.3
N-acetyl-cadaverine	1	1	1	1	1	1	1	1	1
phenylalanine	0.85	0.77	**0.69**	1.15	1.21	1	**1.36**	**1.57**	1.45
N-acetylphenylalanine	0.64	**0.62**	0.88	1.16	1.51	1.39	**1.82**	**2.43**	1.58
phenyllactate (PLA)	0.79	0.8	0.65	0.72	0.98	0.71	0.91	1.22	1.09
phenylacetate	1.03	0.6	0.91	0.61	1.17	0.93	0.6	1.94	1.02

4-hydroxyphenylacetate	0.65	0.78	0.79	0.69	0.94	1.07	1.07	1.19	1.35
phenylacetylglutamine	1.35	0.69	0.8	0.51	0.7	1.09	0.38	1.01	1.35
tyrosine	0.81	**0.62**	**0.62**	1.18	0.96	1.12	**1.46**	**1.53**	**1.8**
N-acetyltyrosine	0.86	0.7	0.67	**9.33**	1.56	1.5	**10.91**	2.23	2.23
tyramine	1	1	1	1	1	1	1	1	1
3-(4-hydroxyphenyl)lactate	0.81	**0.64**	**0.66**	**0.65**	**0.66**	**0.65**	0.8	1.03	1
phenol sulfate	0.66	0.73	0.66	1.34	1.07	1.19	**2.01**	1.47	1.81
p-cresol sulfate	0.91	0.65	0.76	0.61	0.81	0.83	0.68	1.23	1.08
o-cresol sulfate	**3.32**	1.02	1.15	1.07	1.02	0.4	**0.32**	1	**0.35**
3-methoxytyrosine	1.31	0.77	0.83	1.03	0.92	1.02	0.79	1.2	1.23
3-(3-hydroxyphenyl)propionate	0.79	0.89	**0.54**	0.86	1.26	**0.48**	1.08	1.42	0.89
tryptophan	**0.72**	**0.63**	0.75	0.87	0.79	0.82	1.21	1.26	1.09
indolelactate	**0.72**	**0.59**	**0.61**	**0.63**	**0.56**	**0.5**	0.88	0.94	0.82
indoleacetate	1.07	0.75	**0.51**	1.09	0.95	**0.63**	1.02	1.27	1.23
indolepropionate	0.73	**0.59**	**0.38**	**0.65**	0.65	**0.29**	0.89	1.1	0.75
3-indoxyl sulfate	**0.51**	**0.51**	**0.59**	**0.6**	**0.74**	**0.64**	1.17	**1.46**	1.09
kynurenine	**0.77**	**0.64**	**0.69**	**0.71**	**0.62**	**0.58**	0.92	0.97	0.84
kynurenate	1.38	0.87	1.22	0.93	0.86	0.83	0.68	0.98	0.68
serotonin (5HT)	0.71	0.62	**0.54**	**0.41**	**0.3**	**0.31**	0.58	**0.48**	**0.56**
tryptophan betaine	1.03	0.72	**0.84**	**0.59**	**0.65**	**0.69**	**0.57**	0.9	0.82
C-glycosyltryptophan	1.06	0.89	1.09	0.94	0.95	0.99	0.88	1.06	0.91
leucine	1.09	0.78	0.78	1.23	0.85	0.8	1.14	1.09	1.02
4-methyl-2-oxopentanoate	1.14	0.73	0.96	1.01	**0.6**	**0.67**	0.89	0.82	**0.7**
isovalerate	1.37	0.66	1.22	1.58	0.85	1.33	1.15	1.29	1.09
isovalerylcarnitine	0.83	0.75	1.02	**1.89**	1.73	**4.12**	**2.27**	**2.32**	**4.06**
beta-hydroxyisovalerate	1.9	0.73	1.52	**20.74**	0.54	0.51	10.91	0.74	**0.34**
beta-hydroxyisovaleroylcarnitine	1.1	0.9	1.7	0.96	1.02	1.37	0.87	1.13	0.81
3-methylglutarylcarnitine (C6)	0.68	0.79	1.18	0.75	0.51	0.6	1.1	0.65	0.51
alpha-hydroxyisovalerate	0.99	0.82	0.98	1.24	0.76	0.97	1.25	0.93	0.99
isoleucine	1.04	0.75	0.79	1.22	0.91	0.92	1.18	1.22	1.17
3-methyl-2-oxovalerate	1.21	0.77	0.95	1.05	**0.69**	0.67	0.87	0.9	0.71
2-methylbutyrylcarnitine (C5)	0.84	**0.65**	0.79	1.08	**0.62**	0.85	**1.29**	0.96	1.07
tiglyl carnitine	1.41	0.89	2.58	0.72	**0.41**	0.64	**0.51**	0.46	**0.25**
2-hydroxy-3-methylvalerate	1.22	0.69	0.7	1.06	0.78	0.82	0.87	1.13	1.18
3-hydroxy-2-ethylpropionate	1.49	1.23	**1.53**	0.99	1.02	1.02	0.66	0.83	0.67
valine	1.07	0.81	0.85	1.1	**0.78**	**0.79**	1.03	0.97	0.93
3-methyl-2-oxobutyrate	1.26	1.09	1.28	0.78	**0.67**	0.88	**0.62**	**0.62**	**0.69**
isobutyrylcarnitine	0.82	**0.62**	1.39	1.04	0.75	0.8	1.27	1.2	0.58
3-hydroxyisobutyrate	1.03	0.76	1.13	1.14	**0.75**	**0.66**	1.11	0.98	**0.59**
alpha-hydroxyisocaproate	1.43	1.3	1.09	1.1	1.07	1.81	0.77	0.82	1.67
methionine	0.92	0.82	**0.75**	1.02	0.81	0.79	1.11	0.99	1.06

N-acetylmethionine	0.86	1.09	0.81	1.02	**1.92**	1.02	1.18	1.76	1.26
N-formylmethionine	0.97	0.78	0.84	**0.57**	**0.56**	**0.65**	**0.58**	0.72	0.77
homocysteine	1	1	1	1.75	**2.23**	**2.39**	1.75	**2.23**	**2.39**
alpha-ketobutyrate	**2.32**	0.88	**2.48**	0.54	**0.1**	1.24	**0.23**	**0.12**	**0.5**
2-aminobutyrate	**2.21**	1.16	**1.73**	**1.45**	0.82	**1.49**	**0.65**	**0.7**	0.86
2-hydroxybutyrate (AHB)	**2.68**	1.21	**2.79**	1.27	**0.65**	**1.54**	**0.47**	**0.54**	**0.55**
cysteine	1.18	**0.63**	1.08	1.21	1.59	**1.87**	1.02	**2.52**	**1.73**
cystine	0.6	0.45	0.66	0.95	**1.73**	1.24	1.58	**3.85**	1.88
S-methylcysteine	1.24	0.85	0.95	1.36	0.81	1	1.1	0.96	1.06
4-amino-2-hydroxybutyrate	1.11	**0.7**	**1.52**	0.81	**0.66**	**0.76**	0.72	0.94	**0.5**
arginine	0.93	0.79	0.77	0.78	**0.69**	**0.7**	0.84	0.88	0.91
urea	0.94	**0.61**	2.03	0.9	0.71	0.67	0.96	1.17	**0.33**
ornithine	0.59	0.69	0.93	**6.39**	**17.59**	**23.2**	**10.81**	**25.39**	**24.82**
proline	0.81	**0.68**	**0.63**	1.07	0.91	0.83	1.32	1.34	1.32
citrulline	0.9	1.1	1.36	0.86	1.55	1.83	0.95	1.41	1.35
homocitrulline	1.19	0.91	1.23	1.1	**0.68**	0.82	0.92	0.75	0.66
dimethylarginine (SDMA + ADMA)	1.07	1.1	1.07	0.84	0.82	**0.72**	0.78	0.75	**0.68**
N-delta-acetylornithine*	0.6	**0.42**	1.03	0.94	0.85	0.77	1.57	**2.01**	0.75
trans-4-hydroxyproline	1.08	**0.43**	0.55	2.93	0.68	0.54	2.71	1.59	0.99
pro-hydroxy-pro	1.25	0.7	0.76	**4.58**	0.96	1.28	**3.67**	1.38	1.68
creatine	0.76	**0.59**	0.92	1.22	0.91	1.49	**1.61**	**1.54**	**1.61**
creatinine	1.16	0.91	1.01	0.93	0.94	1	**0.8**	1.03	0.98
acisoga	0.98	1	0.86	0.93	0.72	0.98	0.95	0.72	1.13
N-acetylputrescine	0.79	1.01	1.16	0.93	2.11	1.07	1.17	2.09	0.92
4-acetamidobutanoate	1.24	0.78	0.96	1.3	0.86	**0.78**	1.04	1.1	0.81
4-guanidinobutanoate	0.72	**0.5**	0.67	0.9	3.03	1.4	1.25	**6.06**	2.1
glutathione, oxidized (GSSG)	1.01	1.18	0.66	26.34	**23.63**	**101.52**	26.11	**20.03**	**154.42**
cysteine-glutathione disulfide	0.95	1.38	1.28	**5.62**	**4.86**	**10.46**	**5.91**	**3.51**	**8.19**
cys-gly, oxidized	0.68	0.7	1.16	1.17	1.19	**3.63**	1.72	**1.71**	**3.12**
5-oxoproline	0.8	**0.66**	0.71	**4.01**	**7.95**	**8.08**	**4.98**	**12.04**	**11.32**
ophthalmate	0.91	1.69	2.58	2.79	3.63	**8.81**	**3.08**	2.15	**3.41**
gamma-glutamylalanine	**0.58**	**0.64**	0.77	**0.45**	**0.5**	**0.33**	0.77	0.77	**0.43**
gamma-glutamylglutamate	1.24	0.8	0.92	**3.99**	**5.21**	**6.96**	**3.23**	**6.48**	**7.58**
gamma-glutamylglutamine	0.82	0.72	1	0.77	**0.49**	**0.56**	0.93	**0.68**	**0.56**
gamma-glutamylisoleucine*	0.85	**0.56**	0.72	**2.52**	1.71	2.09	**2.95**	**3.06**	**2.91**
gamma-glutamylleucine	0.86	**0.59**	0.75	**1.75**	1.07	1.29	**2.02**	**1.82**	**1.71**
gamma-glutamylmethionine	0.97	0.71	0.91	0.77	**0.48**	**0.47**	0.79	**0.67**	**0.52**
gamma-glutamylphenylalanine	0.84	0.82	0.73	**1.68**	**1.64**	1.69	**1.99**	**2.01**	**2.31**
gamma-glutamylthreonine*	0.88	**0.56**	0.94	**2.24**	**1.81**	**2.31**	**2.55**	**3.22**	**2.46**
gamma-glutamyltyrosine	0.89	**0.57**	**0.62**	**1.76**	1.34	1.59	**1.97**	**2.35**	**2.56**
gamma-glutamylvaline	1.23	**0.6**	1.14	**1.81**	1.21	1.64	1.48	**2.02**	1.44

N-acetylcarnosine	1.06	**0.72**	**0.79**	0.93	**0.78**	**0.68**	0.88	1.08	0.85
alpha-glutamylglutamate	0.61	1.32	**0.25**	1.49	**6.03**	1.55	2.45	**4.57**	**6.09**
alpha-glutamyltyrosine	0.84	0.94	1.16	**0.42**	0.46	0.98	**0.5**	0.48	0.85
aspartylleucine	0.67	0.66	0.91	0.72	**2.8**	1.19	1.08	**4.21**	1.32
aspartylphenylalanine	0.55	0.67	0.98	**0.32**	1.22	0.37	0.58	1.81	0.38
cyclo(glu-glu)	0.64	1.02	0.63	**2.79**	**5.63**	**4.98**	**4.36**	**5.52**	**7.93**
cyclo(leu-pro)	0.57	1.11	**0.46**	0.81	0.76	**0.38**	1.42	0.69	0.83
glycylglycine	0.78	1	0.61	1.09	1.92	1.32	1.4	1.93	2.16
glycylphenylalanine	0.86	1.35	0.95	0.78	**1.96**	0.89	0.9	1.45	0.94
glycylvaline	0.9	0.47	0.37	0.41	1.88	0.42	0.46	**4.01**	1.14
histidyltryptophan	1.4	1.65	0.99	1.37	**2.6**	0.85	0.99	1.58	0.87
isoleucylvaline	1.42	0.73	1.01	**0.31**	**0.28**	**0.3**	**0.22**	**0.39**	**0.3**
leucylalanine	0.77	0.68	1.47	**0.26**	0.64	**0.39**	**0.34**	0.94	**0.26**
phenylalanylglutamate	0.62	1.24	1.37	**4.45**	**10.76**	**4.01**	**7.16**	**8.67**	**2.93**
phenylalanylleucine	0.49	0.48	1.44	0.53	1.79	0.65	1.06	3.74	0.45
phenylalanylphenylalanine	0.58	0.88	0.95	**0.34**	0.76	**0.38**	0.59	0.86	0.4
phenylalanylserine	0.6	1.1	1.3	**0.23**	0.66	0.6	**0.38**	0.61	0.46
phenylalanyltryptophan	0.71	0.68	1.25	**0.36**	**0.44**	0.48	0.51	0.65	0.38
prolylglycine	0.82	0.73	0.8	**4.23**	**3.09**	**7.75**	**5.18**	**4.23**	**9.68**
pyroglutamylglycine	0.68	0.7	0.53	0.91	1.74	0.58	1.33	**2.5**	1.1
pyroglutamylvaline	0.95	0.74	0.8	**81.09**	**111.34**	**180.96**	**84.99**	**150.6**	**226.67**
seryltyrosine	1.19	1.99	1.74	1.54	1.35	1.16	1.29	0.68	0.67
threonylphenylalanine	1	1.06	0.91	**0.61**	**0.62**	**0.49**	**0.61**	**0.59**	**0.53**
tryptophylglutamate	1.52	1.13	0.5	**4.43**	**12.82**	2.1	2.92	**11.31**	**4.25**
tyrosylglutamate	1.05	1.3	1.23	**2.55**	**7.02**	**5.44**	**2.44**	**5.39**	**4.42**
valylarginine	0.79	0.73	1.4	**0.25**	**0.4**	**0.36**	**0.32**	0.54	**0.26**
bradykinin, des-arg(9)	**1.72**	**1.6**	**2.35**	1	1.11	1	**0.58**	0.69	**0.43**
HWESASXX*	**2.74**	0.65	1.05	1.18	0.94	0.87	**0.43**	1.45	0.83
ADSGEGDFXAEGGGVR*	1.05	**7.36**	0.96	1.1	0.6	0.96	1.05	**0.08**	1.01
DSGEGDFXAEGGGVR*	**2.51**	1.23	0.88	1.2	**0.2**	0.76	**0.48**	**0.16**	0.87
ADpSGEGDFXAEGGGVR*	2.86	2.27	0.7	3.54	2.09	**3.38**	1.24	0.92	**4.8**
1,5-anhydroglucitol (1,5-AG)	0.94	0.86	0.93	**0.73**	0.81	**0.63**	**0.77**	0.94	**0.68**
glucose	1.07	0.8	1.18	0.76	**0.53**	0.76	0.71	**0.67**	0.64
2,3-diphosphoglycerate	1	1	1	1	1	1	1	1	1
3-phosphoglycerate	1.03	1.01	0.93	**8.83**	**17.11**	**37.08**	**8.55**	**16.99**	**39.97**
pyruvate	1.05	0.89	1.24	**0.44**	**0.3**	**0.53**	**0.42**	**0.34**	**0.43**
lactate	1.06	1.54	1.51	1.09	**2.44**	1	1.03	1.59	0.66
glycerate	0.99	0.68	0.69	**1.77**	**2.74**	1.07	**1.78**	**4.01**	1.55
ribulose/xylulose 5-phosphate	0.87	1	1.91	**9.26**	**9.94**	**16.01**	**10.7**	**9.94**	**8.37**
ribulose	1.56	0.94	1.09	**11.87**	**7.51**	**5.14**	**7.6**	**8.01**	**4.71**
ribose	0.81	1.05	1.19	**3.98**	**3.07**	1.77	**4.89**	**2.92**	1.49

ribitol	1.01	0.76	0.84	0.8	**2.37**	0.77	0.8	**3.11**	0.92
xylulose	1.41	3.37	1.97	**8.38**	**16.31**	**10.84**	**5.95**	**4.84**	**5.51**
xylonate	0.74	**0.28**	**0.6**	1.4	**0.47**	0.84	1.9	1.72	1.39
xylose	1.05	**0.69**	0.82	0.83	1.12	0.74	0.79	**1.63**	0.91
xylitol	2.23	1.17	1.1	1.14	1.29	2.17	0.51	1.1	1.98
arabinose	0.73	0.97	0.92	**0.4**	**0.49**	0.62	**0.55**	**0.5**	0.67
threitol	0.96	**0.62**	0.93	0.75	0.89	**0.68**	0.79	1.43	0.73
arabitol	0.81	**0.55**	1.42	0.7	0.67	0.84	0.87	1.21	0.59
fucose	1.23	0.78	0.95	**0.41**	0.75	**0.59**	**0.33**	0.97	**0.62**
maltotriose	0.53	0.77	1.71	**1.97**	**6.28**	**4.64**	**3.74**	**8.2**	**2.71**
sucrose	**0.27**	0.4	**0.14**	**2.7**	**35.83**	**6.45**	**9.85**	**89.85**	**44.52**
fructose	1.48	0.61	0.88	0.56	0.48	**0.31**	**0.38**	0.78	**0.35**
sorbitol	0.56	0.53	0.75	**6.39**	**4.52**	**5.64**	**11.31**	**8.49**	**7.56**
mannose	1.59	0.96	1.52	0.79	**0.51**	**0.69**	**0.5**	**0.53**	**0.46**
mannitol	**0.16**	0.63	1.17	0.38	1.04	0.36	2.46	1.65	0.31
glucuronate	0.66	**0.34**	1.1	**0.47**	**0.12**	**0.33**	0.71	**0.36**	**0.3**
N-acetylneuraminate	1.03	0.75	1.33	1.05	1.76	1.54	1.02	**2.34**	1.16
erythronate*	0.88	**0.6**	1.02	**0.74**	0.84	0.87	0.84	**1.42**	0.85
citrate	**1.51**	1.12	1.27	1.16	**1.56**	1.39	0.77	1.38	1.09
alpha-ketoglutarate	0.87	0.54	0.87	0.66	0.61	0.49	0.77	1.12	0.57
succinylcarnitine	0.91	0.95	1.09	1.43	1.41	**2.56**	**1.57**	1.48	**2.36**
fumarate	1.2	0.82	0.82	1.55	**2.71**	**2.04**	1.29	**3.31**	**2.48**
malate	0.79	0.78	0.68	**2.29**	**4.24**	**3.03**	**2.91**	**5.42**	**4.43**
acetylphosphate	1.07	**0.7**	0.89	0.94	0.75	0.93	0.88	1.07	1.05
pyrophosphate (PPi)	2.2	0.88	0.95	2.41	0.67	0.94	1.09	0.75	0.98
phosphate	0.89	0.78	0.91	0.91	0.99	1.22	1.03	1.27	**1.35**
caproate (6:0)	1.12	0.96	0.91	0.8	1.08	0.91	**0.72**	1.12	1.01
heptanoate (7:0)	0.96	1.05	1.24	**17.47**	0.99	1.22	**18.13**	0.94	0.99
caprylate (8:0)	0.94	1.01	0.75	1.22	**1.6**	1.45	1.3	**1.58**	**1.92**
pelargonate (9:0)	0.78	**0.68**	0.91	**0.59**	**0.48**	**0.51**	0.76	**0.7**	**0.56**
caprate (10:0)	0.65	1.02	0.74	0.69	1.3	1.04	1.07	1.27	1.41
10-undecenoate (11:1n1)	1.09	**0.53**	1.1	0.9	**0.63**	1.07	0.82	1.19	0.97
laurate (12:0)	1.16	1.58	2.31	1.56	**3.65**	**2.19**	1.34	**2.31**	0.95
5-dodecenoate (12:1n7)	1.26	1.19	1.39	0.92	1.17	1.31	0.73	0.98	0.95
myristate (14:0)	1.52	1.49	2.01	1.34	2.18	**3.84**	0.88	1.46	1.92
myristoleate (14:1n5)	1.97	**1.99**	**2.16**	1.61	**3.12**	**2.52**	0.82	1.57	1.17
pentadecanoate (15:0)	1.69	1.18	1.13	**3.02**	**3.36**	**2.44**	1.79	2.84	**2.15**
palmitate (16:0)	**1.53**	1.31	**1.62**	1.04	1.08	1.51	**0.68**	0.82	0.93
palmitoleate (16:1n7)	**2.4**	**1.79**	**2.68**	1.54	1.7	**2.23**	0.64	0.95	0.83
margarate (17:0)	**1.83**	1.36	**1.8**	1.32	1.27	**2.21**	0.72	0.93	1.23
10-heptadecenoate (17:1n7)	1.9	1.5	1.93	2.29	**3.8**	**2.94**	1.21	2.53	1.52

stearate (18:0)	1.39	1.22	1.39	0.97	1.05	1.41	**0.7**	0.86	1.01
oleate (18:1n9)	**2.84**	**1.74**	**3.11**	1.57	1.12	**2.18**	**0.55**	0.64	0.7
cis-vaccenate (18:1n7)	**2.18**	**1.52**	**2.07**	1.39	1.13	**1.63**	**0.64**	0.75	0.79
nonadecanoate (19:0)	1.35	1.13	1.09	1.51	1.6	**2.6**	1.12	1.42	**2.39**
10-nonadecenoate (19:1n9)	**2.53**	1.72	**2.08**	1.71	2.09	**2.41**	0.68	1.21	1.16
eicosenoate (20:1n9 or 11)	**2.8**	**1.87**	**2.62**	1.7	1.61	**2.91**	0.61	0.86	1.11
stearidonate (18:4n3)	1.46	1.4	**2.82**	1	1.01	1.32	0.68	0.72	**0.47**
eicosapentaenoate (EPA; 20:5n3)	1.14	1.19	1.39	**0.63**	**0.66**	1.15	**0.55**	**0.56**	0.82
docosapentaenoate (n3 DPA; 22:5n3)	**1.72**	1.59	**2.36**	0.96	0.84	**1.97**	**0.56**	**0.53**	0.83
docosahexaenoate (DHA; 22:6n3)	1.26	1.06	1.2	**0.73**	0.71	1.24	**0.58**	0.67	1.03
linoleate (18:2n6)	**1.93**	**1.46**	**2.09**	0.98	0.91	**1.56**	**0.51**	**0.62**	0.74
linolenate [alpha or gamma; (18:3n3 or 6)]	**2.81**	**1.6**	**2.67**	0.84	0.75	1.4	**0.3**	**0.47**	**0.53**
dihomo-linolenate (20:3n3 or n6)	1.24	1.09	**1.62**	0.76	0.75	1.22	**0.61**	0.69	0.75
arachidonate (20:4n6)	1.16	0.93	0.96	0.84	1.08	1.2	0.72	1.16	1.25
adrenate (22:4n6)	0.96	1.12	0.65	**0.53**	0.75	**0.55**	**0.56**	0.67	0.85
docosapentaenoate (n6 DPA; 22:5n6)	1.54	1.08	1.22	0.85	0.8	1.13	**0.55**	0.74	0.93
docosadienoate (22:2n6)	1.84	1.68	1.76	2.33	**3.17**	**3.28**	1.27	1.88	1.86
dihomo-linoleate (20:2n6)	**2.33**	**1.77**	**2.64**	1.43	1.57	**2.8**	0.61	0.89	1.06
13-methylmyristic acid	1.05	0.93	1.28	1.05	1.06	2.15	1	1.13	1.68
15-methylpalmitate (isobar with 2-methylpalmitate)	1.58	1.29	1.33	2.12	**4.94**	**2.26**	1.34	3.84	1.7
17-methylstearate	**1.92**	1.38	1.17	1.71	**2.36**	**2.23**	0.89	1.71	1.92
2-hydroxyglutarate	**0.74**	1.09	**0.63**	1.04	**1.77**	1.1	**1.4**	**1.62**	**1.74**
azelate (nonanedioate)	**3.4**	**4.6**	1.43	**3.96**	**6**	**21.88**	1.16	1.31	**15.32**
sebacate (decanedioate)	**2.39**	1.73	0.94	**5.28**	**3.68**	**6.45**	2.21	2.13	**6.88**
undecanedioate	1.34	**0.55**	0.61	**3.02**	**2.92**	**3.61**	**2.25**	**5.35**	**5.93**
1,11-undecanedicarboxylate	0.9	0.83	0.64	**4.51**	**8.2**	**26.79**	**4.99**	**9.82**	**41.8**
dodecanedioate	1.76	1.17	0.96	1.76	1.93	1.42	1	1.64	1.48
tetradecanedioate	**1.89**	1.48	1.15	1.1	0.9	0.8	**0.58**	0.6	0.7
hexadecanedioate	**1.76**	1.64	1.24	1.17	1.13	1.18	0.66	0.69	0.96
octadecanedioate	**2.09**	1.56	1.29	1.51	1.22	1.42	**0.72**	0.78	1.1
3-carboxy-4-methyl-5-propyl-2-furanpropanoate (CMPF)	1.14	**0.64**	0.85	**0.43**	**0.44**	0.64	**0.38**	0.7	0.75
palmitate, methyl ester	0.97	**0.52**	0.92	**0.42**	**0.35**	**0.61**	**0.44**	0.67	**0.66**
n-Butyl Oleate	0.92	1.4	1.26	0.88	1.3	1.23	0.95	0.92	0.98
2-aminoheptanoate	0.71	**0.59**	**0.41**	0.72	0.61	**0.56**	1.01	1.04	1.35
2-aminooctanoate	0.82	**0.62**	**0.54**	0.79	0.74	**0.59**	0.97	1.18	1.09
butyrylcarnitine	0.79	**0.64**	0.75	1.07	0.81	0.82	1.35	1.26	1.09
propionylcarnitine	**0.58**	**0.52**	0.93	1.13	0.8	1.2	**1.92**	**1.54**	**1.29**
acetylcarnitine	**1.74**	1.29	**1.63**	**1.71**	1.16	**1.55**	0.98	0.9	0.95
hydroxybutyrylcarnitine*	**8.65**	**3.87**	**6.95**	**5.99**	**2.83**	**4.89**	**0.69**	0.73	0.7
hexanoylcarnitine	0.87	0.91	1.12	1.17	0.88	1.6	1.34	0.97	1.42
octanoylcarnitine	**0.53**	**0.54**	0.8	0.9	**0.41**	1.43	**1.68**	0.76	**1.79**

decanoylcarnitine	**0.48**	**0.57**	0.68	0.93	0.7	1.27	**1.95**	1.24	**1.88**
cis-4-decenoyl carnitine	**0.57**	**0.59**	**0.61**	0.78	**0.67**	1.19	1.37	1.15	**1.94**
laurylcarnitine	0.79	**0.43**	0.97	0.9	0.53	1	1.14	1.23	1.03
palmitoylcarnitine	1.25	0.91	1.36	0.9	0.87	1.5	0.72	0.96	1.1
stearoylcarnitine	1.09	1.26	1.09	0.8	0.8	1.31	0.73	0.63	1.2
oleoylcarnitine	1.2	0.71	1.19	0.81	0.92	1.73	0.68	1.29	1.45
deoxycarnitine	0.93	**0.7**	0.95	1.03	0.96	**1.28**	1.11	**1.36**	**1.34**
carnitine	1.03	0.99	1.11	0.99	0.86	0.9	0.95	0.88	**0.81**
3-dehydrocarnitine	0.94	**0.81**	1.05	1.02	**0.73**	1.03	1.08	0.9	0.98
acetoacetate	**13.88**	**5.66**	**8.71**	2.06	**0.46**	3.58	**0.15**	**0.08**	**0.41**
3-hydroxybutyrate (BHBA)	**18.77**	**5.79**	**11.25**	**9.44**	2.5	**5.87**	**0.5**	**0.43**	0.52
2-hydroxydecanoate	0.81	**0.36**	**0.52**	0.61	0.61	1.46	0.75	**1.69**	**2.81**
2-hydroxypalmitate	1.08	0.93	0.95	0.76	0.97	1.53	0.7	1.04	1.61
2-hydroxystearate	0.87	0.73	0.76	0.64	1.01	1.35	0.73	1.38	1.77
3-hydroxypropanoate	1.07	0.86	0.9	1.21	0.9	0.95	1.14	1.04	1.05
3-hydroxyoctanoate	1.43	0.76	0.39	0.93	0.61	0.59	0.65	0.81	1.49
3-hydroxydecanoate	1.22	0.69	0.46	0.96	0.72	0.77	0.78	1.04	1.68
13-HODE + 9-HODE	**4.57**	**2.46**	**2.32**	1.34	**2.49**	**3.74**	**0.29**	1.01	1.61
prostaglandin E2	1	1	1	1	1.26	**1.53**	1	1.26	**1.53**
12-HETE	0.78	1.26	0.86	1.39	**13.96**	2.22	1.79	**11.06**	2.58
myo-inositol	0.99	0.69	1.44	0.95	0.88	1.28	0.95	1.27	0.89
chiro-inositol	1.1	1.49	0.58	0.49	0.7	3.1	0.45	0.47	5.39
scyllo-inositol	0.93	**0.65**	2.03	0.95	0.7	1.76	1.02	1.08	0.87
inositol 1-phosphate (I1P)	0.76	0.65	**0.8**	0.75	**0.59**	0.78	0.99	0.91	0.97
choline	**0.69**	**0.69**	**0.68**	1.01	1.07	0.95	**1.47**	**1.55**	1.39
glycerophosphorylcholine (GPC)	0.87	1.04	0.8	0.96	**1.77**	**2.05**	1.11	**1.71**	**2.58**
1-myristoylglycerophosphocholine (14:0)	**0.33**	**0.32**	0.82	**0.27**	**0.31**	**0.28**	0.81	0.99	**0.34**
2-myristoylglycerophosphocholine*	**0.23**	**0.49**	**0.71**	**0.21**	**0.66**	**0.33**	0.93	1.36	0.47
1-pentadecanoylglycerophosphocholine (15:0)*	0.8	**0.37**	0.9	0.61	**0.42**	0.7	0.76	1.13	0.78
1-palmitoylglycerophosphocholine (16:0)	0.79	0.63	0.8	**0.55**	0.61	**0.6**	0.7	0.98	**0.74**
2-palmitoylglycerophosphocholine*	0.77	**0.44**	0.75	**0.45**	**0.43**	**0.53**	0.58	0.98	**0.71**
1-palmitoleoylglycerophosphocholine (16:1)*	**0.6**	**0.44**	**0.67**	**0.37**	**0.39**	**0.4**	**0.61**	0.89	**0.6**
1-margaroylglycerophosphocholine (17:0)	0.71	**0.36**	0.68	**0.43**	**0.44**	**0.7**	0.61	1.21	1.03
1-stearoylglycerophosphocholine (18:0)	0.48	0.61	0.74	**0.33**	0.59	**0.46**	0.7	0.97	**0.62**
2-stearoylglycerophosphocholine*	**0.29**	**0.57**	0.68	**0.32**	0.61	**0.45**	1.11	1.06	0.66
1-oleoylglycerophosphocholine (18:1)	0.78	0.55	0.75	**0.37**	**0.42**	**0.51**	**0.47**	0.76	**0.68**
2-oleoylglycerophosphocholine*	0.89	0.68	0.9	**0.53**	**0.55**	0.75	**0.6**	0.81	0.83
1-linoleoylglycerophosphocholine (18:2n6)	0.65	**0.5**	0.65	**0.31**	**0.3**	**0.39**	**0.47**	0.59	**0.61**
2-linoleoylglycerophosphocholine*	0.69	**0.4**	**0.58**	**0.36**	**0.31**	**0.39**	**0.53**	0.76	**0.66**
1-linolenoylglycerophosphocholine (18:3n3)*	**0.4**	**0.51**	**0.81**	**0.18**	**0.32**	**0.34**	**0.46**	0.63	0.41
1-dihomo-linoleoylglycerophosphocholine (20:2n6)*	**0.48**	**0.5**	0.65	**0.31**	**0.34**	**0.44**	0.64	0.68	0.67

1-eicosatrienoylglycerophosphocholine (20:3)*	**0.57**	**0.35**	0.78	**0.2**	**0.16**	**0.32**	**0.35**	**0.47**	**0.41**
1-arachidonoylglycerophosphocholine (20:4n6)*	1.37	0.64	0.92	**0.46**	**0.37**	**0.49**	**0.33**	0.58	**0.53**
2-arachidonoylglycerophosphocholine*	1.21	0.75	1.04	0.67	0.75	0.61	0.55	1.01	0.59
1-eicosapentaenoylglycerophosphocholine (20:5n3)*	0.62	**0.5**	1.13	**0.15**	**0.2**	**0.5**	**0.24**	**0.4**	**0.45**
1-docosapentaenoylglycerophosphocholine (22:5n3)*	1.02	0.84	0.93	**0.54**	**0.45**	0.77	0.53	0.54	0.83
1-docosahexaenoylglycerophosphocholine (22:6n3)*	1.36	0.83	1.04	**0.53**	**0.49**	**0.89**	**0.39**	**0.58**	**0.85**
1-palmitoylplasmenylethanolamine*	**0.41**	**0.55**	0.69	0.72	**2.02**	1.15	**1.77**	**3.68**	1.66
1-stearoylplasmenylethanolamine*	0.97	0.94	0.43	1.54	3	0.49	1.58	**3.2**	1.16
1-palmitoylglycerophosphoethanolamine	0.77	0.71	1.01	0.74	0.97	0.97	0.96	1.36	0.96
2-palmitoylglycerophosphoethanolamine*	0.79	0.84	1.14	0.73	1.3	1.03	0.92	**1.54**	0.9
1-stearoylglycerophosphoethanolamine	0.69	0.78	0.91	0.72	1.26	0.72	1.04	**1.62**	0.79
1-oleoylglycerophosphoethanolamine	0.69	**0.62**	0.81	**0.45**	**0.68**	**0.56**	**0.66**	1.09	**0.69**
2-oleoylglycerophosphoethanolamine*	**0.72**	**0.57**	0.87	**0.46**	**0.7**	**0.64**	**0.63**	1.24	0.73
1-linoleoylglycerophosphoethanolamine*	**0.51**	**0.52**	**0.6**	**0.39**	**0.46**	**0.41**	0.76	0.88	**0.68**
2-linoleoylglycerophosphoethanolamine*	**0.48**	**0.59**	**0.63**	**0.4**	**0.61**	**0.52**	0.83	1.04	0.82
1-arachidonoylglycerophosphoethanolamine*	0.92	**0.81**	0.87	**0.74**	**0.76**	0.78	**0.81**	0.93	0.9
2-arachidonoylglycerophosphoethanolamine*	0.9	0.99	0.93	0.82	1.01	0.93	0.91	1.02	1
2-docosahexaenoylglycerophosphoethanolamine*	1.1	0.72	0.59	1.26	0.73	0.59	1.14	1	0.99
1-docosahexaenoylglycerophosphoethanolamine*	1.11	1.05	1.13	0.88	0.99	1.2	0.8	0.94	1.06
1-palmitoylglycerophosphoinositol*	**0.52**	**0.53**	0.9	**0.3**	**0.39**	**0.54**	0.58	0.73	0.6
1-stearoylglycerophosphoinositol	0.65	0.66	0.71	**0.29**	**0.38**	**0.57**	**0.44**	0.58	0.8
2-stearoylglycerophosphoinositol*	0.68	0.69	0.77	0.49	**0.26**	**0.55**	0.72	**0.38**	0.71
1-arachidonoylglycerophosphoinositol*	1.03	0.72	1.12	**0.47**	**0.44**	**0.69**	**0.45**	**0.61**	**0.61**
1-palmitoylglycerophosphate	1.07	0.6	0.67	0.81	1.22	1.02	0.76	**2.02**	1.51
1-arachidonoylglyercophosphate	1.07	0.55	0.89	0.63	1	0.89	0.59	1.83	1
glycerol	**1.98**	**1.99**	**1.85**	**3.25**	**8.37**	**3.51**	1.64	**4.21**	1.9
glycerol 3-phosphate (G3P)	0.67	**0.56**	**0.71**	**0.61**	1.27	0.97	0.91	**2.27**	**1.37**
1-myristoylglycerol (1-monomyristin)	1.16	1.4	**4.99**	**0.59**	0.61	**0.42**	**0.51**	0.44	**0.08**
1-palmitoylglycerol (1-monopalmitin)	**1.78**	**1.77**	**2.72**	1.22	1.61	0.8	0.68	0.91	**0.29**
2-palmitoylglycerol (2-monopalmitin)	0.91	1.06	1.34	1	1.04	1.02	1.1	0.98	0.76
1-stearoylglycerol (1-monostearin)	**0.71**	0.84	1.16	**0.69**	0.98	0.88	0.98	1.16	0.76
1-oleoylglycerol (1-monoolein)	**7.26**	**3.84**	**5.8**	2.05	0.94	0.76	**0.28**	**0.25**	**0.13**
1-linoleoylglycerol (1-monolinolein)	**6.48**	**4.75**	**5.77**	1.87	1.15	0.73	**0.29**	**0.24**	**0.13**
1-linolenoylglycerol	**9.97**	**7.16**	**7.06**	0.93	0.67	**0.38**	**0.09**	**0.09**	**0.05**
1,2-dipalmitoylglycerol	1.07	0.44	2.17	0.36	1.25	1.05	0.33	**2.85**	0.49
palmitoyl sphingomyelin	0.87	**0.67**	0.84	0.82	0.96	1.02	0.94	**1.43**	1.21
stearoyl sphingomyelin	1.49	0.98	1.18	**1.64**	**1.51**	**2.14**	1.1	**1.54**	**1.8**
sphingosine 1-phosphate	0.73	**0.49**	0.75	**0.63**	**0.47**	**0.61**	0.86	0.95	0.82
sphingosine	0.88	0.76	**0.21**	1.08	0.96	**0.24**	1.22	1.26	1.16
lathosterol	0.77	0.82	1.25	1.09	1.33	**1.87**	1.42	1.63	**1.49**
cholesterol	0.81	**0.66**	0.93	**0.79**	0.86	1.02	0.98	**1.3**	1.1

7-beta-hydroxycholesterol	**0.48**	0.86	0.6	0.78	1.15	1.02	1.63	1.33	1.71
7-alpha-hydroxy-3-oxo-4-cholestenoate (7-Hoca)	**0.65**	**0.67**	0.97	**0.63**	0.82	0.94	0.96	1.23	0.97
cholestanol	0.77	1.04	1.14	0.8	1.23	0.96	1.04	1.18	0.84
beta-sitosterol	**0.54**	**0.59**	1	**0.54**	**0.59**	1.06	1	1	1.06
campesterol	**0.61**	**0.43**	0.72	0.69	0.65	**0.4**	1.13	1.51	0.56
pregnenolone sulfate	0.75	0.75	**0.64**	0.79	0.84	**0.61**	1.05	1.12	0.94
21-hydroxypregnenolone disulfate	1.5	1.06	0.74	1.5	0.78	0.97	1	0.74	1.32
5alpha-pregnan-3beta,20alpha-diol disulfate	1.06	2.09	0.91	0.66	2.57	0.95	0.63	1.23	1.04
5alpha-pregnan-3alpha,20beta-diol disulfate 1*	0.63	2.56	0.88	0.58	2.99	1.01	0.92	1.17	1.14
pregnen-diol disulfate*	1.42	1.04	1.08	**1.53**	0.93	1.45	1.08	0.89	1.34
pregn steroid monosulfate*	0.98	0.75	0.72	0.98	0.92	0.84	1	1.24	1.18
pregnanediol-3-glucuronide	0.68	1.75	0.94	0.44	1.09	0.85	0.64	0.62	0.9
cortisol	1.11	1.14	0.74	1.46	7.27	0.72	1.32	6.4	0.97
cortisone	0.97	0.74	0.67	1.2	0.74	1.14	1.23	1.01	**1.71**
dehydroisoandrosterone sulfate (DHEA-S)	1.04	0.69	0.62	0.9	**0.79**	0.71	0.86	1.14	1.14
epiandrosterone sulfate	0.95	0.63	0.59	0.74	**0.69**	**0.79**	0.78	1.09	1.34
androsterone sulfate	1.4	0.79	0.69	1	**0.78**	0.94	0.71	0.99	1.36
4-androsten-3beta,17beta-diol disulfate (1)*	1.1	0.69	0.76	0.95	**0.61**	1.46	0.87	0.88	1.92
4-androsten-3beta,17beta-diol disulfate (2)*	1.39	0.85	0.83	1.16	0.76	1	0.83	0.9	1.22
5alpha-androstan-3beta,17beta-diol disulfate	1.08	0.71	0.73	0.88	**0.63**	1.14	0.81	0.89	1.55
andro steroid monosulfate 2*	**1.64**	1.05	0.84	**1.4**	0.93	0.92	0.85	0.88	**1.1**
cholate	**0.04**	**0.34**	0.44	0.15	0.98	0.74	**3.47**	2.93	1.67
glycocholate	0.4	**0.17**	**0.4**	0.7	0.67	1.78	1.76	**3.84**	4.41
taurocholate	0.82	**0.17**	0.31	1.08	0.36	3.64	1.31	2.05	**11.78**
glycochenodeoxycholate	0.63	**0.29**	**0.2**	0.87	0.47	1.18	1.39	1.59	**6.06**
taurochenodeoxycholate	1.71	0.32	0.45	1.15	0.3	2.77	0.67	0.94	6.19
glycodeoxycholate	1.41	**0.34**	**0.29**	0.76	0.87	**0.6**	0.54	**2.56**	2.1
taurodeoxycholate	1.65	0.2	**0.29**	1.13	0.3	1.53	0.68	1.53	5.23
glycolithocholate sulfate*	**2.96**	0.71	**0.23**	**3.56**	1.7	0.76	1.2	2.38	**3.29**
taurolithocholate 3-sulfate	**4.28**	0.33	0.73	**3.56**	0.56	1.2	0.83	1.72	1.64
glycoursodeoxycholate	1.27	**0.3**	**0.42**	0.7	0.61	**0.61**	0.55	2.03	1.45
hyocholate	0.71	**0.5**	0.79	0.71	**0.57**	**0.56**	1.01	1.13	0.7
glycohyocholate	1.13	**0.37**	0.74	1.55	0.59	1.4	1.38	1.58	**1.9**
glycocholenate sulfate*	1.06	0.9	**0.57**	1.35	0.86	0.98	1.28	0.95	**1.7**
taurocholenate sulfate*	**2.69**	0.85	1.42	**2.06**	0.68	**2.05**	0.77	0.8	1.44
inosine	0.86	1.51	**1.72**	**3.04**	**3.52**	**2.3**	**3.53**	2.34	1.34
hypoxanthine	0.74	0.94	0.7	**3.03**	**7.25**	**2.07**	**4.09**	**7.73**	**2.97**
xanthine	0.79	0.84	0.87	1.24	**1.49**	1.11	**1.57**	**1.77**	1.28
xanthosine	1.32	1.43	1.19	1.22	**2.3**	**2.53**	0.92	1.61	**2.13**
2'-deoxyinosine	1.78	5.3	1	1.83	**4.2**	**8.54**	1.03	0.79	**8.54**
urate	1.12	0.92	0.94	0.91	0.76	**0.72**	0.81	0.82	**0.77**

allantoin	**0.43**	**0.38**	**0.31**	**9.42**	**21.79**	**10.08**	**21.79**	**57.52**	**32.06**
adenosine 5'-monophosphate (AMP)	1	21.91	1	26.36	**32.11**	80.16	26.36	1.47	80.16
adenosine	1.21	17.92	1.12	3.14	2.05	1.76	2.6	0.11	1.57
N1-methyladenosine	1	0.89	0.91	**0.64**	**0.53**	**0.48**	**0.64**	**0.59**	**0.53**
N6-carbamoylthreonyladenosine	0.99	1.02	1.03	1.08	1.08	1.24	1.09	1.06	1.21
guanosine	1.11	0.94	1.32	0.68	0.41	0.58	0.61	0.43	0.44
7-methylguanine	0.7	0.9	0.87	1.01	0.88	0.75	1.45	0.98	0.87
N1-methylguanosine	1.73	2.07	0.55	1.28	1.54	1.06	0.74	0.74	**1.94**
N2,N2-dimethylguanosine	1.02	1.31	1.24	0.96	0.88	1.02	0.94	0.67	0.82
uridine	0.87	**0.81**	1.02	0.94	1.04	0.83	1.08	**1.29**	0.81
pseudouridine	1.04	**0.79**	1.09	0.96	0.78	0.81	0.92	0.99	**0.75**
5-methyluridine (ribothymidine)	0.89	0.79	1.03	0.82	0.75	1.02	0.92	0.95	0.99
beta-alanine	0.56	**0.37**	0.68	0.61	0.98	**0.45**	1.1	**2.62**	0.66
N-acetyl-beta-alanine	0.81	0.69	0.76	1.37	1.15	1.42	**1.68**	**1.67**	**1.86**
methylphosphate	0.84	**0.73**	**0.72**	0.84	0.93	0.98	0.99	1.26	**1.37**
nicotinamide	0.67	1.72	1.32	**7.51**	**7.69**	**6.9**	**11.29**	**4.47**	**5.24**
N1-Methyl-2-pyridone-5-carboxamide	2.13	0.84	**1.98**	1.05	**0.52**	**0.46**	**0.49**	0.61	**0.23**
riboflavin (Vitamin B2)	1.45	0.7	0.9	1.06	**0.55**	**0.57**	0.73	0.78	0.63
pantothenate	**2.53**	**1.67**	1.26	**1.52**	1.11	1.32	**0.6**	0.67	1.04
gulono-1,4-lactone	1.16	0.77	0.85	**1.82**	**2.66**	1.56	1.57	**3.47**	**1.84**
ascorbate (Vitamin C)	1.72	0.42	1.1	**0.08**	**0.02**	**0.07**	**0.05**	**0.05**	**0.06**
threonate	**1.4**	0.66	1.47	0.81	0.69	0.85	**0.58**	1.05	**0.58**
arabonate	1	0.69	1.2	0.93	0.67	1.08	0.94	0.97	0.9
oxalate (ethanedioate)	**1.62**	0.76	**1.83**	0.99	0.56	1.08	**0.61**	0.73	0.59
alpha-tocopherol	0.84	**0.64**	1.08	**0.52**	**0.6**	**0.58**	**0.62**	0.93	**0.54**
beta-tocopherol	**0.46**	0.71	1.02	**0.42**	0.8	**0.43**	0.91	1.14	**0.43**
gamma-tocopherol	**0.23**	**0.6**	0.69	**0.39**	0.64	**0.61**	**1.71**	1.07	0.88
gamma-CEHC	**0.63**	**0.45**	**0.38**	**0.38**	**0.24**	**0.17**	0.6	**0.54**	**0.45**
gamma-CEHC glucuronide*	**0.54**	**0.45**	**0.36**	**0.39**	**0.28**	**0.28**	0.71	0.62	0.8
alpha-CEHC glucuronide*	0.88	1.18	1.3	0.58	0.63	0.64	0.66	**0.53**	**0.49**
heme	2.15	5.17	3.85	**108.58**	**94.24**	**104.2**	**50.62**	**18.24**	**27.04**
bilirubin (Z,Z)	0.73	0.73	1.01	**0.29**	**0.1**	**0.3**	**0.4**	**0.14**	**0.3**
bilirubin (E,E)*	1.31	0.62	1.8	**0.37**	**0.18**	**0.44**	**0.28**	**0.29**	**0.24**
bilirubin (E,Z or Z,E)*	1.06	1.29	1.66	1.03	2.36	3.56	0.97	1.83	2.15
biliverdin	**2.3**	1.07	**2.32**	**3.03**	2.43	**5.87**	1.32	2.26	**2.53**
I-urobilinogen	1.61	0.62	0.7	1.71	0.58	1.4	1.06	0.93	2
L-urobilin	0.66	2.09	0.44	0.63	0.54	1.59	0.94	**0.26**	**3.59**
pyridoxate	2.04	0.88	0.88	1.6	0.49	0.57	0.79	0.55	0.64
hippurate	0.53	**0.4**	**0.3**	0.83	0.69	1.3	1.58	1.73	**4.29**
2-hydroxyhippurate (salicylurate)	0.17	1.5	**0.1**	0.14	0.82	**0.03**	0.81	0.55	0.32
3-hydroxyhippurate	**0.18**	**0.3**	**0.27**	**0.28**	**0.28**	0.64	1.56	0.92	**2.32**

4-hydroxyhippurate	1.15	0.66	**0.41**	**0.54**	0.82	0.64	0.47	1.24	1.56
benzoate	0.82	**0.78**	0.91	**0.67**	**0.67**	**0.66**	0.81	0.86	**0.72**
catechol sulfate	**0.59**	0.93	**0.69**	**0.59**	**0.34**	**0.49**	1.01	**0.37**	0.72
O-methylcatechol sulfate	0.48	0.71	0.62	0.52	**0.2**	**0.87**	1.09	0.29	1.4
3-methyl catechol sulfate (1)	**0.15**	**0.21**	**0.02**	**0.48**	**0.12**	**0.14**	3.09	0.58	**5.77**
3-methyl catechol sulfate (2)	0.84	1	**0.32**	0.93	1	**0.33**	1.11	1	1.02
4-methylcatechol sulfate	0.9	0.57	0.75	0.66	0.95	0.97	0.73	1.65	1.29
3-ethylphenylsulfate*	1	0.62	**0.45**	1	0.62	**0.45**	1	1	1
4-ethylphenylsulfate	1.54	**0.38**	1.39	0.48	**0.24**	0.38	0.32	0.64	0.27
4-vinylphenol sulfate	**0.2**	**0.11**	**0.13**	**0.16**	1.03	**0.5**	0.8	**9.22**	3.8
caffeine	0.43	0.29	0.12	0.44	**0.1**	0.13	1.02	0.36	1.1
paraxanthine	0.88	0.47	0.27	0.43	**0.17**	0.24	0.49	**0.36**	0.91
theobromine	**1.28**	**0.23**	1.82	1.13	**0.25**	0.72	0.88	1.12	0.39
theophylline	0.96	**0.32**	0.4	0.7	**0.19**	0.36	0.72	0.6	0.9
7-methylurate	1.59	0.41	0.81	**0.61**	**0.24**	0.44	0.38	0.59	0.55
1,3-dimethylurate	1.24	0.46	0.58	0.6	**0.32**	**0.47**	0.49	0.69	0.81
1,7-dimethylurate	1.41	**0.24**	0.69	**0.36**	**0.08**	0.95	0.25	0.32	1.38
1,3,7-trimethylurate	0.37	0.36	0.14	0.4	**0.19**	0.19	1.07	0.53	1.37
1-methylxanthine	0.93	0.71	0.55	0.62	0.67	0.52	0.66	0.94	0.96
3-methylxanthine	1.81	0.83	1.28	0.88	**0.45**	0.64	0.49	0.54	0.5
7-methylxanthine	1.85	0.67	1.24	0.77	0.53	0.71	0.42	0.79	0.57
5-acetylamino-6-amino-3-methyluracil	1.6	0.63	0.82	0.78	**0.4**	0.66	0.48	0.63	0.81
cotinine	1	1	**0.83**	1	1	**0.83**	1	1	1
2-piperidinone	**0.24**	0.28	1.12	1.32	0.59	1.27	5.59	2.11	1.14
1,6-anhydroglucose	0.9	0.92	1.58	1.12	0.54	0.76	1.25	0.59	0.48
gluconate	0.81	**0.54**	1.17	0.83	1.25	1.5	1.02	**2.31**	1.28
ergothioneine	0.73	0.84	1.01	1.17	1.55	**3.41**	1.61	**1.84**	**3.39**
erythritol	0.92	0.69	0.85	0.83	1.21	0.77	0.9	1.77	0.91
homostachydrine*	0.71	0.8	0.95	**0.67**	**0.3**	**0.49**	0.95	**0.38**	**0.51**
N-(2-furoyl)glycine	0.19	1	**0.64**	1	1	**0.08**	5.25	1	0.13
piperine	**0.15**	**0.09**	**0.36**	**0.29**	**0.2**	**0.44**	**1.88**	2.07	1.24
2-furoic acid	0.58	0.65	**0.21**	0.79	0.49	0.27	1.36	0.76	1.28
quinate	**0.25**	0.22	**0.04**	0.5	0.66	0.28	2.04	3.07	**6.86**
saccharin	2.3	1.65	0.09	**8.01**	**6.92**	**1.17**	**3.48**	**4.19**	**12.65**
stachydrine	0.62	0.36	0.57	0.21	**0.05**	1.43	0.34	0.14	2.53
thymol sulfate	**0.6**	**0.09**	0.68	**0.67**	0.59	**0.25**	1.11	**6.67**	**0.36**
tribuloside	1	1	1	1	1	1	1	1	1
2-hydroxyacetaminophen sulfate*	0.49	1	0.4	0.67	8.1	0.33	1.36	8.1	0.84
2-methoxyacetaminophen sulfate*	0.48	1	0.42	0.62	3.88	0.95	1.31	3.88	2.27
3-(cystein-S-yl)acetaminophen*	0.75	1	0.69	0.75	1.38	0.99	1	1.38	1.43
3-(N-acetyl-L-cystein-S-yl) acetaminophen*	1	1	1	1	1	1	1	1	1

4-acetaminophen sulfate	0.9	1.3	0.31	0.79	21.59	0.14	0.88	16.55	0.46
4-acetamidophenol	0.55	1	0.43	0.66	4.33	0.79	1.2	4.33	1.81
4-acetamidophenylglucuronide	0.21	1.21	0.37	0.6	7.41	0.6	2.83	6.14	1.61
ibuprofen acyl glucuronide	1	**0.9**	1	1	**0.9**	1	1	1	1
ibuprofen	**0.83**	0.91	1	**0.83**	0.91	1	1	1	1
2-hydroxyibuprofen	1	**0.8**	1	1	**0.8**	1	1	1	1
carboxyibuprofen	**0.9**	0.95	1	**0.9**	0.95	1	1	1	1
atenolol	1	1	0.75	1	1.44	0.72	1	1.44	0.96
desmethylnaproxen sulfate*	0.97	**0.75**	1	0.97	0.88	1	1	1.17	1
O-desmethylvenlafaxine	**0.94**	1	1	**0.94**	1	1	1	1	1
escitalopram	**0.85**	1	1	**0.85**	1	1	1	1	1
fluoxetine	**1.18**	1	1	0.98	1	1	**0.83**	1	1
gabapentin	1	1	**0.48**	1	1	0.54	1	1	1.12
hydrochlorothiazide	1.05	1	0.94	0.96	1	**0.8**	0.91	1	0.85
hydroquinone sulfate	1	1	0.91	1	1	1.06	1	1	**1.17**
hydroxypioglitazone (M-IV)	1	1	1	1	1	1	1	1	1
ketopioglitazone	1	1	1	1	1	1	1	1	1
lidocaine	**8.5**	**18.75**	**12.61**	1	1	1	**0.12**	**0.05**	**0.08**
metformin	1	**0.21**	**0.23**	1	**0.29**	**0.33**	1	1.37	1.43
metoprolol	0.97	1	1	**0.88**	1	1	**0.91**	1	1
metoprolol acid metabolite*	**1.11**	1	1	**1.08**	1	1	0.98	1	1
naproxen	0.49	0.33	0.86	**0.03**	1.25	0.86	0.07	3.82	1
norfluoxetine	**1.09**	1	1	1.02	1	1	0.94	1	1
omeprazole	1	**1.13**	1	1	1	1	1	**0.89**	1
pantoprazole	0.1	0.52	11.84	**6.24**	2.6	**50.56**	**64.39**	5.02	4.27
pioglitazone*	1	1	1	1	1	1	1	1	1
salicylate	0.39	1.37	0.1	0.24	1.68	0.13	0.63	1.23	1.33
1,2-propanediol	1.7	3.17	0.81	**6.4**	**4.06**	2.52	**3.77**	1.28	**3.09**
2-aminophenol sulfate	**0.16**	**0.18**	1.06	**0.3**	0.34	**0.25**	1.83	1.87	0.23
bisphenol A monosulfate	1.35	1.62	0.98	1.67	**2.94**	2.67	1.24	1.81	2.71
2-ethylhexanoate	0.76	**0.53**	0.98	**0.41**	**0.32**	**0.48**	**0.54**	0.6	**0.49**
2-hydroxyisobutyrate	1.41	0.54	7.86	0.88	9.48	0.69	0.62	17.58	**0.09**
dexpanthenol	1	1	1	1	**4.61**	1	1	**4.61**	1
glycerol 2-phosphate	0.92	0.78	**0.68**	0.8	**1.58**	0.99	0.88	**2.03**	**1.45**
glycolate (hydroxyacetate)	1.24	0.74	0.79	1.59	1.26	1	1.28	**1.71**	1.27

* Green and red shaded cells indicate p≤0.05 (green indicates that the mean values are significantly higher for that comparison; red - values significantly lower). Light green and light red shaded cells indicate 0.05<p<0.10 (light green indicates that the mean values trend higher for that comparison; light red - values trend lower).

Table A2. A Global Metabolomic Study: Analytical Technique of Detection (Platform) and The Percentage of Detection in a Given Group or Time Point (% filled values) for all 476 compounds detected.										
Biochemical Name	Platform	% Filled Values								
		GB T2D BL	GB T2D Pre-Op	GB T2D Post-Op	SG T2D BL	SG T2D Pre-Op	SG T2D Post-Op	SG Non-T2D BL	SG Non-T2D Pre-Op	SG Non-T2D Post-Op
glycine	GC/MS	100	100	100	100	100	100	100	100	100
N-acetylglycine	GC/MS	100	100	100	80	100	100	100	100	100
dimethylglycine	GC/MS	100	100	100	100	100	100	100	100	100
betaine	LC/MS pos	100	100	100	100	100	100	100	100	100
serine	GC/MS	100	100	100	100	100	100	100	100	100
N-acetylserine	GC/MS	100	100	100	100	80	100	100	100	100
beta-hydroxypyruvate	GC/MS	60	80	40	80	60	40	60	80	60
threonine	LC/MS pos	100	100	100	100	100	100	100	100	100
N-acetylthreonine	LC/MS neg	100	100	100	100	100	100	100	100	100
homoserine	GC/MS	60	40	20	60	40	20	20	60	0
alanine	GC/MS	100	100	100	100	100	100	100	100	100
N-acetylalanine	LC/MS neg	100	100	100	100	100	100	100	100	100
aspartate	GC/MS	100	100	100	100	100	100	100	100	100
asparagine	GC/MS	100	100	100	100	100	100	100	100	100
glutamate	LC/MS pos	100	100	100	100	100	100	100	100	100
glutamine	LC/MS pos	100	100	100	100	100	100	100	100	100
pyroglutamine*	LC/MS pos	100	100	100	100	100	100	100	100	100
histidine	LC/MS neg	100	100	100	100	100	100	100	100	100
1-methylhistidine	GC/MS	60	40	60	20	0	60	40	40	60
3-methylhistidine	LC/MS neg	100	100	100	100	60	100	100	100	100
trans-urocanate	LC/MS pos	100	100	100	80	100	100	100	100	100
cis-urocanate	LC/MS neg	0	20	100	0	40	100	40	60	100
imidazole propionate	LC/MS pos	40	20	100	20	20	100	20	0	80
lysine	LC/MS pos	100	100	100	100	100	100	100	100	100
glutarylcarnitine (C5)	LC/MS pos	100	100	100	100	100	100	100	100	100
pipecolate	LC/MS pos	100	100	100	100	100	100	100	100	100
N-acetyl-cadaverine	LC/MS pos	0	0	0	0	0	20	0	0	0
phenylalanine	LC/MS pos	100	100	100	100	100	100	100	100	100
N-acetylphenylalanine	LC/MS neg	100	80	80	100	80	100	100	40	80
phenyllactate (PLA)	LC/MS neg	100	100	100	100	80	60	100	80	100

phenylacetate	GC/MS	40	60	80	60	20	80	60	60	20
4-hydroxyphenylacetate	GC/MS	80	60	40	60	40	60	60	40	60
phenylacetylglutamine	LC/MS pos	100	100	100	100	100	100	100	100	100
tyrosine	LC/MS pos	100	100	100	100	100	100	100	100	100
N-acetyltyrosine	LC/MS neg	20	0	60	60	0	20	60	40	80
tyramine	LC/MS pos	0	0	0	0	0	20	0	0	0
3-(4-hydroxyphenyl)lactate	LC/MS neg	100	100	100	100	100	100	100	100	100
phenol sulfate	LC/MS neg	100	100	100	100	100	100	100	100	100
p-cresol sulfate	LC/MS neg	100	100	100	100	80	100	100	100	100
o-cresol sulfate	LC/MS neg	80	80	60	0	20	20	20	80	20
3-methoxytyrosine	LC/MS pos	100	100	100	100	100	100	100	100	100
3-(3-hydroxyphenyl)propionate	LC/MS neg	60	20	0	20	0	20	40	0	40
tryptophan	LC/MS pos	100	100	100	100	100	100	100	100	100
indolelactate	GC/MS	100	100	100	100	100	100	100	100	100
indoleacetate	LC/MS pos	100	100	100	100	100	100	100	100	100
indolepropionate	LC/MS neg	100	60	80	100	60	80	100	100	100
3-indoxyl sulfate	LC/MS neg	100	100	100	100	100	100	100	100	100
kynurenine	LC/MS pos	100	100	100	100	100	100	100	100	100
kynurenate	LC/MS neg	100	100	100	100	80	100	100	100	100
serotonin (5HT)	LC/MS pos	100	100	80	100	100	80	100	80	80
tryptophan betaine	LC/MS pos	100	100	100	100	100	100	100	100	100
C-glycosyltryptophan	LC/MS pos	100	100	100	100	100	100	100	100	100
leucine	LC/MS pos	100	100	100	100	100	100	100	100	100
4-methyl-2-oxopentanoate	LC/MS neg	100	100	100	100	100	100	100	100	100
isovalerate	LC/MS neg	80	100	80	100	100	100	80	80	100
isovalerylcarnitine	LC/MS pos	100	100	100	100	80	100	100	100	100
beta-hydroxyisovalerate	LC/MS neg	100	100	60	100	100	100	100	100	100
beta-hydroxyisovaleroylcarnitine	LC/MS pos	100	100	100	100	100	100	100	100	100
3-methylglutarylcarnitine (C6)	LC/MS pos	100	100	80	100	100	80	100	100	100
alpha-hydroxyisovalerate	LC/MS neg	100	100	100	100	100	100	100	100	100
isoleucine	LC/MS pos	100	100	100	100	100	100	100	100	100
3-methyl-2-oxovalerate	LC/MS neg	100	100	100	100	100	100	100	100	100
2-methylbutyrylcarnitine (C5)	LC/MS pos	100	100	100	100	80	100	100	100	100
tiglyl carnitine	LC/MS pos	100	80	80	100	60	60	100	100	100
2-hydroxy-3-methylvalerate	LC/MS	60	60	40	80	60	60	80	60	60

	neg									
3-hydroxy-2-ethylpropionate	GC/MS	80	100	100	80	100	80	100	100	100
valine	LC/MS pos	100	100	100	100	100	100	100	100	100
3-methyl-2-oxobutyrate	LC/MS neg	100	100	100	100	100	100	100	100	100
isobutyrylcarnitine	LC/MS pos	100	100	100	100	100	100	100	100	100
3-hydroxyisobutyrate	GC/MS	100	100	100	100	100	100	100	100	100
alpha-hydroxyisocaproate	GC/MS	20	20	40	20	60	40	60	60	60
methionine	LC/MS pos	100	100	100	100	100	100	100	100	100
N-acetylmethionine	LC/MS neg	100	100	100	100	80	100	100	100	100
N-formylmethionine	LC/MS neg	100	100	100	100	100	80	100	100	80
homocysteine	GC/MS	20	0	60	0	0	40	0	0	20
alpha-ketobutyrate	GC/MS	40	100	60	100	100	40	80	100	60
2-aminobutyrate	LC/MS pos	100	100	100	100	100	100	100	100	100
2-hydroxybutyrate (AHB)	GC/MS	100	100	100	100	100	100	100	100	100
cysteine	GC/MS	100	100	100	100	100	100	100	100	100
cystine	GC/MS	100	100	100	100	40	100	100	60	100
S-methylcysteine	LC/MS pos	100	100	100	100	100	100	100	100	100
4-amino-2-hydroxybutyrate	GC/MS	100	100	100	100	80	100	100	100	100
arginine	LC/MS pos	100	100	80	100	100	100	100	100	100
urea	GC/MS	100	100	100	100	80	100	100	100	100
ornithine	GC/MS	100	100	100	100	100	100	100	100	100
proline	LC/MS pos	100	100	100	100	100	100	100	100	100
citrulline	LC/MS pos	100	100	100	100	100	100	100	100	100
homocitrulline	LC/MS pos	100	100	100	100	100	100	100	100	100
dimethylarginine (SDMA + ADMA)	LC/MS pos	100	100	100	100	100	100	100	100	100
N-delta-acetylornithine*	LC/MS pos	100	100	100	100	100	100	100	100	100
trans-4-hydroxyproline	GC/MS	100	100	100	100	100	100	100	100	100
pro-hydroxy-pro	LC/MS pos	100	100	100	100	100	100	100	100	100
creatine	LC/MS pos	100	100	100	100	100	100	100	100	100
creatinine	LC/MS pos	100	100	100	100	100	100	100	100	100
acisoga	LC/MS pos	100	100	100	100	100	100	100	100	100
N-acetylputrescine	LC/MS pos	80	80	80	80	80	60	80	60	80
4-acetamidobutanoate	LC/MS pos	100	100	100	100	100	100	100	100	100
4-guanidinobutanoate	LC/MS pos	80	40	100	100	40	80	60	20	60
glutathione, oxidized (GSSG)	LC/MS pos	100	60	80	60	60	80	80	60	60
cysteine-glutathione disulfide	LC/MS pos	80	100	100	100	100	100	100	100	100

cys-gly, oxidized	LC/MS neg	60	80	100	100	80	100	100	80	100
5-oxoproline	LC/MS neg	100	100	100	100	100	100	100	100	100
ophthalmate	LC/MS pos	0	40	100	20	60	60	40	20	60
gamma-glutamylalanine	LC/MS pos	100	100	100	100	100	80	100	100	100
gamma-glutamylglutamate	LC/MS pos	100	80	100	100	80	100	100	100	100
gamma-glutamylglutamine	LC/MS pos	100	100	80	100	100	100	100	100	100
gamma-glutamylisoleucine*	LC/MS pos	100	100	100	100	100	100	100	80	100
gamma-glutamylleucine	LC/MS pos	100	100	100	100	100	100	100	100	100
gamma-glutamylmethionine	LC/MS pos	100	100	60	100	100	60	100	100	100
gamma-glutamylphenylalanine	LC/MS pos	100	100	100	100	100	100	100	100	100
gamma-glutamylthreonine*	LC/MS pos	100	100	100	100	100	100	100	100	100
gamma-glutamyltyrosine	LC/MS pos	100	100	100	100	100	100	100	100	100
gamma-glutamylvaline	LC/MS pos	100	100	100	100	100	100	100	100	100
N-acetylcarnosine	LC/MS pos	100	100	100	100	100	100	100	100	100
alpha-glutamylglutamate	LC/MS pos	40	0	80	0	20	100	20	0	40
alpha-glutamyltyrosine	LC/MS pos	40	60	40	100	80	40	100	100	80
aspartylleucine	LC/MS pos	80	80	80	80	40	80	100	80	100
aspartylphenylalanine	LC/MS pos	100	100	100	100	80	100	100	100	100
cyclo(glu-glu)	LC/MS pos	60	20	100	40	20	100	60	0	100
cyclo(leu-pro)	LC/MS pos	80	40	40	60	40	40	40	40	60
glycylglycine	GC/MS	100	100	100	60	60	80	100	80	100
glycylphenylalanine	LC/MS neg	100	80	60	20	40	80	100	100	80
glycylvaline	LC/MS pos	100	80	100	100	100	100	100	100	100
histidyltryptophan	LC/MS pos	100	100	100	100	100	100	100	100	100
isoleucylvaline	LC/MS pos	60	80	20	100	100	40	80	80	20
leucylalanine	LC/MS pos	100	100	80	100	80	100	100	100	100
phenylalanylglutamate	LC/MS pos	80	60	100	60	40	100	100	60	100
phenylalanylleucine	LC/MS neg	100	80	80	100	100	100	100	100	100
phenylalanylphenylalanine	LC/MS pos	100	100	100	100	100	100	100	100	100
phenylalanylserine	LC/MS pos	60	80	80	80	80	60	100	100	100
phenylalanyltryptophan	LC/MS pos	100	80	80	100	100	60	100	100	100
prolylglycine	LC/MS pos	80	100	100	80	40	100	80	20	100
pyroglutamylglycine	LC/MS neg	80	60	100	100	60	100	100	100	100
pyroglutamylvaline	LC/MS neg	20	20	100	60	0	100	20	20	100

seryltyrosine	LC/MS pos	80	100	80	100	80	60	100	100	100
threonylphenylalanine	LC/MS pos	100	80	60	80	80	20	100	100	60
tryptophylglutamate	LC/MS pos	80	60	100	20	40	100	60	100	100
tyrosylglutamate	LC/MS pos	40	60	60	0	20	100	60	60	100
valylarginine	LC/MS neg	80	80	60	100	80	40	100	100	80
bradykinin, des-arg(9)	LC/MS neg	0	40	0	20	60	20	0	60	0
HWESASXX*	LC/MS pos	100	100	80	100	100	100	100	100	80
ADSGEGDFXAEGGGVR*	LC/MS pos	100	100	100	100	100	100	100	100	100
DSGEGDFXAEGGGVR*	LC/MS pos	100	100	100	100	100	100	80	100	80
ADpSGEGDFXAEGGGVR*	LC/MS pos	40	60	100	100	100	80	80	100	100
1,5-anhydroglucitol (1,5-AG)	GC/MS	100	100	100	100	100	100	100	100	100
glucose	GC/MS	100	100	100	100	100	100	100	100	100
2,3-diphosphoglycerate	LC/MS neg	0	0	20	0	0	0	0	0	0
3-phosphoglycerate	GC/MS	40	20	80	0	20	80	20	20	60
pyruvate	GC/MS	100	100	80	100	100	80	100	100	100
lactate	GC/MS	100	100	100	100	100	100	100	100	100
glycerate	GC/MS	100	100	100	100	100	100	100	100	100
ribulose/xylulose 5-phosphate	GC/MS	0	20	80	0	20	60	20	20	60
ribulose	GC/MS	80	80	80	40	60	80	20	60	100
ribose	GC/MS	100	100	100	100	60	80	100	100	100
ribitol	GC/MS	100	100	80	100	80	100	100	100	100
xylulose	GC/MS	20	60	80	20	80	80	60	60	100
xylonate	GC/MS	100	80	100	80	20	80	80	40	100
xylose	GC/MS	100	100	100	80	60	100	80	80	80
xylitol	GC/MS	80	100	80	60	60	60	60	80	60
arabinose	GC/MS	60	100	60	100	100	40	80	80	60
threitol	GC/MS	100	100	80	100	100	100	100	100	100
arabitol	GC/MS	80	100	100	100	100	100	100	100	100
fucose	GC/MS	100	100	100	100	80	80	100	100	60
maltotriose	GC/MS	40	60	100	80	40	80	80	40	100
sucrose	LC/MS neg	80	40	100	80	20	100	80	20	100
fructose	GC/MS	100	100	100	100	100	100	100	100	100
sorbitol	GC/MS	80	80	100	100	80	100	60	60	100
mannose	GC/MS	100	100	100	100	100	80	100	100	100
mannitol	GC/MS	100	100	100	100	80	100	80	80	100
glucuronate	GC/MS	100	100	80	100	80	60	100	80	80
N-acetylneuraminate	GC/MS	100	100	100	100	80	100	100	100	80
erythronate*	GC/MS	100	100	100	100	100	100	100	100	100
citrate	GC/MS	100	100	100	100	100	100	100	100	100

alpha-ketoglutarate	GC/MS	80	80	80	80	80	80	100	100	80
succinylcarnitine	LC/MS pos	100	100	100	100	100	100	100	100	100
fumarate	GC/MS	100	100	100	100	100	100	100	100	100
malate	GC/MS	100	100	100	100	100	100	100	100	100
acetylphosphate	GC/MS	100	100	100	100	100	100	100	100	100
pyrophosphate (PPi)	GC/MS	100	100	100	100	80	100	80	100	100
phosphate	GC/MS	100	100	100	100	100	100	100	100	100
caproate (6:0)	LC/MS neg	100	100	100	100	80	100	100	100	100
heptanoate (7:0)	LC/MS neg	80	80	80	80	80	100	80	60	80
caprylate (8:0)	LC/MS neg	100	100	100	100	100	100	100	100	100
pelargonate (9:0)	GC/MS	100	100	100	100	100	100	100	100	100
caprate (10:0)	LC/MS neg	100	100	100	100	100	100	100	100	100
10-undecenoate (11:1n1)	LC/MS neg	100	100	100	100	100	100	100	100	100
laurate (12:0)	LC/MS neg	100	100	100	100	100	100	100	100	100
5-dodecenoate (12:1n7)	LC/MS neg	100	100	100	100	100	100	100	100	100
myristate (14:0)	LC/MS neg	100	100	100	100	100	100	100	100	100
myristoleate (14:1n5)	LC/MS neg	100	100	100	100	100	100	100	100	100
pentadecanoate (15:0)	GC/MS	100	100	100	100	100	100	100	100	100
palmitate (16:0)	LC/MS neg	100	100	100	100	100	100	100	100	100
palmitoleate (16:1n7)	LC/MS neg	100	100	100	100	100	100	100	100	100
margarate (17:0)	LC/MS neg	100	100	100	100	100	100	100	100	100
10-heptadecenoate (17:1n7)	LC/MS neg	100	100	100	100	100	100	100	100	100
stearate (18:0)	LC/MS neg	100	100	100	100	100	100	100	100	100
oleate (18:1n9)	GC/MS	100	100	100	100	100	100	100	100	100
cis-vaccenate (18:1n7)	GC/MS	100	100	100	100	100	100	100	100	100
nonadecanoate (19:0)	LC/MS neg	100	100	100	100	100	100	100	100	100
10-nonadecenoate (19:1n9)	LC/MS neg	100	100	100	100	100	100	100	100	100
eicosenoate (20:1n9 or 11)	LC/MS neg	100	100	100	100	100	100	100	100	100
stearidonate (18:4n3)	LC/MS neg	100	100	80	100	100	100	100	100	80
eicosapentaenoate (EPA; 20:5n3)	LC/MS neg	100	100	100	100	100	100	100	100	100
docosapentaenoate (n3 DPA; 22:5n3)	LC/MS neg	100	100	100	100	100	100	100	100	100
docosahexaenoate (DHA; 22:6n3)	LC/MS neg	100	100	100	100	100	100	100	100	100
linoleate (18:2n6)	LC/MS neg	100	100	100	100	100	100	100	100	100
linolenate [alpha or gamma; (18:3n3 or 6)]	LC/MS neg	100	100	100	100	100	100	100	100	100
dihomo-linolenate (20:3n3 or n6)	LC/MS neg	100	100	100	100	100	100	100	100	100
arachidonate (20:4n6)	LC/MS neg	100	100	100	100	100	100	100	100	100

adrenate (22:4n6)	LC/MS neg	100	100	100	100	100	100	100	100	100
docosapentaenoate (n6 DPA; 22:5n6)	LC/MS neg	100	100	100	100	100	100	100	100	100
docosadienoate (22:2n6)	LC/MS neg	100	100	100	100	100	100	100	100	100
dihomo-linoleate (20:2n6)	LC/MS neg	100	100	100	100	100	100	100	100	100
13-methylmyristic acid	GC/MS	20	20	20	20	40	60	20	20	40
15-methylpalmitate (isobar with 2-methylpalmitate)	LC/MS neg	100	100	100	100	100	100	100	100	100
17-methylstearate	LC/MS neg	100	100	100	100	100	100	100	100	100
2-hydroxyglutarate	GC/MS	100	100	100	80	60	100	100	40	100
azelate (nonanedioate)	LC/MS neg	80	100	100	60	100	100	100	100	100
sebacate (decanedioate)	LC/MS neg	60	100	100	40	60	100	20	100	80
undecanedioate	LC/MS neg	60	60	100	60	0	100	0	40	100
1,11-undecanedicarboxylate	LC/MS neg	80	60	100	40	40	100	100	80	100
dodecanedioate	LC/MS neg	100	100	100	100	80	100	100	100	100
tetradecanedioate	LC/MS neg	100	100	100	100	100	100	100	100	100
hexadecanedioate	LC/MS neg	100	100	100	100	100	100	100	100	100
octadecanedioate	LC/MS neg	100	100	100	100	100	100	100	100	100
3-carboxy-4-methyl-5-propyl-2-furanpropanoate (CMPF)	LC/MS neg	100	100	100	100	80	100	100	100	100
palmitate, methyl ester	GC/MS	100	100	80	100	100	100	100	100	100
n-Butyl Oleate	GC/MS	20	40	20	20	40	40	60	40	20
2-aminoheptanoate	LC/MS pos	100	80	80	100	100	100	100	100	80
2-aminooctanoate	LC/MS pos	100	100	100	100	100	100	100	100	100
butyrylcarnitine	LC/MS pos	100	100	100	100	100	100	100	100	100
propionylcarnitine	LC/MS pos	100	100	100	100	100	100	100	100	100
acetylcarnitine	LC/MS pos	100	100	100	100	100	100	100	100	100
hydroxybutyrylcarnitine*	LC/MS pos	100	100	100	100	100	100	100	100	100
hexanoylcarnitine	LC/MS pos	100	100	100	100	100	100	100	100	100
octanoylcarnitine	LC/MS pos	100	100	100	100	100	100	100	100	100
decanoylcarnitine	LC/MS pos	100	100	100	100	100	100	100	100	100
cis-4-decenoyl carnitine	LC/MS pos	100	100	100	100	100	100	100	100	100
laurylcarnitine	LC/MS pos	80	100	60	100	100	100	60	60	100
palmitoylcarnitine	LC/MS pos	100	100	80	100	100	100	100	100	100
stearoylcarnitine	LC/MS pos	100	80	100	80	100	100	100	80	80
oleoylcarnitine	LC/MS pos	100	100	80	100	100	100	100	100	100
deoxycarnitine	LC/MS pos	100	100	100	100	100	100	100	100	100
carnitine	LC/MS pos	100	100	100	100	100	100	100	100	100

3-dehydrocarnitine	LC/MS pos	100	100	100	100	100	100	100	100	100
acetoacetate	GC/MS	100	100	100	100	100	100	100	100	100
3-hydroxybutyrate (BHBA)	GC/MS	100	100	100	100	100	100	100	100	100
2-hydroxydecanoate	LC/MS neg	100	100	100	100	80	100	100	100	100
2-hydroxypalmitate	LC/MS neg	100	100	100	100	100	100	100	100	100
2-hydroxystearate	LC/MS neg	100	100	100	100	100	100	100	100	100
3-hydroxypropanoate	GC/MS	100	100	100	100	100	100	100	100	100
3-hydroxyoctanoate	LC/MS neg	80	100	80	100	80	100	100	100	100
3-hydroxydecanoate	LC/MS neg	100	100	100	100	100	100	100	100	100
13-HODE + 9-HODE	LC/MS neg	100	100	100	100	100	100	100	100	100
prostaglandin E2	LC/MS neg	0	0	20	0	0	40	0	0	0
12-HETE	LC/MS neg	100	80	100	80	100	100	100	100	100
myo-inositol	GC/MS	100	100	100	100	100	100	100	100	100
chiro-inositol	GC/MS	40	40	40	40	60	60	60	20	0
scyllo-inositol	GC/MS	100	100	80	100	80	100	100	100	100
inositol 1-phosphate (I1P)	GC/MS	100	100	100	100	100	80	100	100	100
choline	LC/MS pos	100	100	100	100	100	100	100	100	100
glycerophosphorylcholine (GPC)	LC/MS pos	80	80	100	100	80	100	100	100	100
1-myristoylglycerophosphocholine (14:0)	LC/MS pos	100	100	60	100	80	80	100	80	100
2-myristoylglycerophosphocholine*	LC/MS neg	100	40	40	80	40	80	100	80	60
1-pentadecanoylglycerophosphocholine (15:0)*	LC/MS pos	100	100	80	100	40	60	100	100	100
1-palmitoylglycerophosphocholine (16:0)	LC/MS pos	100	100	100	100	100	100	100	100	100
2-palmitoylglycerophosphocholine*	LC/MS pos	100	100	100	100	100	100	100	100	100
1-palmitoleoylglycerophosphocholine (16:1)*	LC/MS pos	100	100	60	100	100	100	100	100	100
1-margaroylglycerophosphocholine (17:0)	LC/MS pos	100	100	40	100	60	100	100	100	80
1-stearoylglycerophosphocholine (18:0)	LC/MS pos	100	100	100	100	100	100	100	100	100
2-stearoylglycerophosphocholine*	LC/MS pos	100	100	80	100	100	100	100	100	100
1-oleoylglycerophosphocholine (18:1)	LC/MS pos	100	100	100	100	100	100	100	100	100
2-oleoylglycerophosphocholine*	LC/MS pos	100	100	60	100	100	80	100	100	100
1-linoleoylglycerophosphocholine (18:2n6)	LC/MS pos	100	100	100	100	100	100	100	100	100
2-linoleoylglycerophosphocholine*	LC/MS pos	100	100	80	100	100	100	100	100	100
1-linolenoylglycerophosphocholine (18:3n3)*	LC/MS pos	80	40	40	80	40	0	80	60	0
1-dihomo-linoleoylglycerophosphocholine (20:2n6)*	LC/MS pos	100	40	20	100	40	40	100	80	40
1-eicosatrienoylglycerophosphocholine (20:3)*	LC/MS pos	100	100	60	100	100	100	100	100	100
1-arachidonoylglycerophosphocholine (20:4n6)*	LC/MS pos	100	100	100	100	100	100	100	100	100

2-arachidonoylglycerophosphocholine*	LC/MS pos	100	100	80	60	100	100	80	100	80
1-eicosapentaenoylglycerophosphocholine (20:5n3)*	LC/MS pos	100	100	60	100	60	20	100	80	40
1-docosapentaenoylglycerophosphocholine (22:5n3)*	LC/MS pos	100	100	80	80	80	80	100	80	100
1-docosahexaenoylglycerophosphocholine (22:6n3)*	LC/MS pos	100	100	100	100	100	100	100	100	100
1-palmitoylplasmenylethanolamine*	LC/MS neg	100	100	100	100	100	100	100	100	100
1-stearoylplasmenylethanolamine*	LC/MS neg	100	100	100	100	100	100	100	100	100
1-palmitoylglycerophosphoethanolamine	LC/MS neg	100	100	100	100	100	100	100	100	100
2-palmitoylglycerophosphoethanolamine*	LC/MS neg	100	100	100	100	100	100	100	100	100
1-stearoylglycerophosphoethanolamine	LC/MS neg	100	100	100	100	100	100	100	100	100
1-oleoylglycerophosphoethanolamine	LC/MS neg	100	100	100	100	100	100	100	100	100
2-oleoylglycerophosphoethanolamine*	LC/MS neg	100	100	100	100	100	100	100	100	100
1-linoleoylglycerophosphoethanolamine*	LC/MS neg	100	100	100	100	100	100	100	100	100
2-linoleoylglycerophosphoethanolamine*	LC/MS neg	100	100	100	100	80	100	100	100	100
1-arachidonoylglycerophosphoethanolamine*	LC/MS neg	100	100	100	100	100	100	100	100	100
2-arachidonoylglycerophosphoethanolamine*	LC/MS neg	100	100	100	100	100	100	100	100	100
2-docosahexaenoylglycerophosphoethanolamine*	LC/MS pos	100	80	80	60	20	80	60	80	100
1-docosahexaenoylglycerophosphoethanolamine*	LC/MS neg	100	100	100	100	100	100	100	100	100
1-palmitoylglycerophosphoinositol*	LC/MS neg	100	80	80	100	80	100	100	100	100
1-stearoylglycerophosphoinositol	LC/MS neg	100	100	100	100	100	100	100	100	100
2-stearoylglycerophosphoinositol*	LC/MS neg	100	100	80	80	80	40	80	80	80
1-arachidonoylglycerophosphoinositol*	LC/MS neg	100	100	100	100	100	100	100	100	100
1-palmitoylglycerophosphate	LC/MS neg	100	100	80	100	100	100	100	100	100
1-arachidonoylglyercophosphate	LC/MS neg	100	100	100	100	100	100	100	100	100
glycerol	GC/MS	100	100	100	100	100	100	100	100	100
glycerol 3-phosphate (G3P)	GC/MS	100	100	100	100	100	100	100	100	100
1-myristoylglycerol (1-monomyristin)	LC/MS neg	100	100	60	100	80	60	100	100	60
1-palmitoylglycerol (1-monopalmitin)	GC/MS	100	100	100	100	100	100	100	100	100
2-palmitoylglycerol (2-monopalmitin)	GC/MS	0	20	20	20	80	40	20	20	40
1-stearoylglycerol (1-monostearin)	GC/MS	100	100	100	100	100	100	100	100	100
1-oleoylglycerol (1-monoolein)	GC/MS	100	100	80	60	80	80	100	100	100
1-linoleoylglycerol (1-monolinolein)	LC/MS neg	100	100	100	100	100	100	100	100	100
1-linolenoylglycerol	LC/MS neg	100	100	60	60	100	60	100	100	60
1,2-dipalmitoylglycerol	GC/MS	20	60	60	80	40	100	80	60	60
palmitoyl sphingomyelin	GC/MS	100	100	100	100	100	100	100	100	100
stearoyl sphingomyelin	GC/MS	100	100	100	100	100	100	100	100	100
sphingosine 1-phosphate	LC/MS	100	100	60	100	100	80	100	100	60

	pos									
sphingosine	LC/MS pos	100	60	80	60	40	80	60	20	80
lathosterol	GC/MS	80	80	100	60	40	60	100	60	100
cholesterol	GC/MS	100	100	100	100	100	100	100	100	100
7-beta-hydroxycholesterol	GC/MS	100	80	80	60	100	100	100	60	100
7-alpha-hydroxy-3-oxo-4-cholestenoate (7-Hoca)	LC/MS neg	100	100	100	100	100	100	100	100	100
cholestanol	GC/MS	100	100	100	100	100	100	100	100	100
beta-sitosterol	GC/MS	0	0	20	60	0	0	80	20	0
campesterol	GC/MS	80	60	20	60	20	40	80	40	80
pregnenolone sulfate	LC/MS neg	100	100	100	100	100	100	100	100	100
21-hydroxypregnenolone disulfate	LC/MS neg	100	100	100	100	80	100	100	100	100
5alpha-pregnan-3beta,20alpha-diol disulfate	LC/MS neg	100	100	100	100	100	100	100	100	100
5alpha-pregnan-3alpha,20beta-diol disulfate 1*	LC/MS neg	40	60	80	40	40	60	40	60	60
pregnen-diol disulfate*	LC/MS neg	100	100	100	100	100	100	100	100	100
pregn steroid monosulfate*	LC/MS neg	100	100	100	100	100	100	100	100	100
pregnanediol-3-glucuronide	LC/MS neg	80	100	100	100	80	100	100	100	80
cortisol	LC/MS pos	100	100	100	100	100	100	100	100	100
cortisone	LC/MS pos	100	100	100	100	100	80	100	100	100
dehydroisoandrosterone sulfate (DHEA-S)	LC/MS neg	100	100	100	100	100	100	100	100	100
epiandrosterone sulfate	LC/MS neg	100	100	100	100	100	100	100	100	100
androsterone sulfate	LC/MS neg	100	100	100	100	100	100	100	100	100
4-androsten-3beta,17beta-diol disulfate (1)*	LC/MS neg	100	100	100	100	100	100	100	100	100
4-androsten-3beta,17beta-diol disulfate (2)*	LC/MS neg	100	100	100	100	100	100	100	100	100
5alpha-androstan-3beta,17beta-diol disulfate	LC/MS neg	100	100	100	100	100	100	100	100	100
andro steroid monosulfate 2*	LC/MS neg	100	100	100	100	100	100	100	100	100
cholate	LC/MS neg	100	100	100	100	80	80	80	40	100
glycocholate	LC/MS neg	100	60	80	100	40	100	80	40	60
taurocholate	LC/MS neg	100	100	100	100	80	100	80	60	80
glycochenodeoxycholate	LC/MS neg	100	80	100	100	100	100	100	100	100
taurochenodeoxycholate	LC/MS neg	100	100	100	100	80	80	80	100	100
glycodeoxycholate	LC/MS neg	100	80	100	100	60	100	100	100	100
taurodeoxycholate	LC/MS neg	100	80	100	100	80	100	100	100	100
glycolithocholate sulfate*	LC/MS neg	100	100	100	100	100	100	100	100	100
taurolithocholate 3-sulfate	LC/MS neg	100	100	100	100	100	100	100	100	100
glycoursodeoxycholate	LC/MS neg	100	100	100	100	100	100	100	80	100

hyocholate	LC/MS neg	100	100	100	100	100	100	100	100	100
glycohyocholate	LC/MS neg	100	80	100	80	40	60	100	80	100
glycocholenate sulfate*	LC/MS neg	100	100	100	100	100	100	100	80	100
taurocholenate sulfate*	LC/MS neg	100	100	100	100	100	100	100	100	100
inosine	LC/MS neg	100	100	100	100	100	100	100	100	100
hypoxanthine	LC/MS neg	100	100	100	100	100	100	100	100	100
xanthine	LC/MS pos	100	100	100	100	100	100	100	100	100
xanthosine	LC/MS pos	0	40	60	0	60	80	20	20	60
2'-deoxyinosine	LC/MS neg	0	0	80	0	20	80	0	40	60
urate	LC/MS neg	100	100	100	100	100	100	100	100	100
allantoin	GC/MS	100	80	100	100	40	100	100	80	100
adenosine 5'-monophosphate (AMP)	LC/MS pos	20	0	20	0	40	40	0	0	20
adenosine	LC/MS pos	40	60	80	40	40	60	60	40	60
N1-methyladenosine	LC/MS pos	100	100	100	100	100	100	100	100	100
N6-carbamoylthreonyladenosine	LC/MS pos	100	100	80	100	100	80	100	80	100
guanosine	LC/MS neg	80	100	80	100	100	100	80	80	80
7-methylguanine	LC/MS pos	100	100	100	100	100	100	100	100	100
N1-methylguanosine	LC/MS pos	100	60	100	80	80	100	20	60	40
N2,N2-dimethylguanosine	LC/MS pos	100	100	80	100	100	80	100	80	100
uridine	LC/MS neg	100	100	100	100	100	100	100	100	100
pseudouridine	LC/MS neg	100	100	100	100	100	100	100	100	100
5-methyluridine (ribothymidine)	LC/MS neg	80	100	100	100	80	80	100	100	100
beta-alanine	GC/MS	100	100	80	100	60	100	100	100	100
N-acetyl-beta-alanine	LC/MS pos	100	100	100	100	100	100	100	80	100
methylphosphate	GC/MS	100	80	100	100	100	100	100	100	100
nicotinamide	LC/MS pos	100	100	100	100	100	100	100	100	100
N1-Methyl-2-pyridone-5-carboxamide	LC/MS pos	100	100	60	100	100	100	100	100	100
riboflavin (Vitamin B2)	LC/MS pos	80	80	40	80	60	40	60	60	40
pantothenate	LC/MS pos	100	100	80	100	100	100	100	100	100
gulono-1,4-lactone	GC/MS	100	100	100	100	100	100	80	100	100
ascorbate (Vitamin C)	GC/MS	100	80	60	100	100	40	100	100	80
threonate	GC/MS	100	100	100	100	100	100	100	100	100
arabonate	GC/MS	100	100	100	100	100	100	100	100	100
oxalate (ethanedioate)	GC/MS	100	80	100	100	100	100	100	100	100
alpha-tocopherol	GC/MS	100	100	100	100	100	100	100	100	100
beta-tocopherol	GC/MS	80	60	0	80	80	60	100	100	80

gamma-tocopherol	GC/MS	100	100	100	100	100	100	100	100	100
gamma-CEHC	LC/MS neg	100	100	80	100	80	80	100	100	80
gamma-CEHC glucuronide*	LC/MS neg	100	60	60	80	80	20	100	20	0
alpha-CEHC glucuronide*	LC/MS neg	60	80	0	60	60	0	20	60	0
heme	LC/MS pos	100	100	100	100	80	100	100	80	100
bilirubin (Z,Z)	LC/MS neg	100	100	80	100	100	60	100	100	80
bilirubin (E,E)*	LC/MS pos	100	100	80	100	80	100	100	100	100
bilirubin (E,Z or Z,E)*	LC/MS pos	80	80	40	60	60	40	80	80	60
biliverdin	LC/MS neg	80	100	100	100	100	100	100	100	100
I-urobilinogen	LC/MS neg	80	100	80	100	80	40	80	80	100
L-urobilin	LC/MS pos	60	60	80	80	60	40	60	40	40
pyridoxate	LC/MS neg	100	100	100	100	100	100	100	100	100
hippurate	LC/MS neg	100	100	100	100	100	100	100	100	100
2-hydroxyhippurate (salicylurate)	LC/MS neg	100	60	80	60	60	80	60	80	80
3-hydroxyhippurate	LC/MS neg	100	20	80	60	20	40	100	0	60
4-hydroxyhippurate	LC/MS neg	100	100	100	100	100	100	100	80	80
benzoate	GC/MS	100	100	100	100	100	100	100	100	100
catechol sulfate	LC/MS neg	100	100	100	100	100	100	100	100	100
O-methylcatechol sulfate	LC/MS neg	100	80	80	100	80	100	80	100	80
3-methyl catechol sulfate (1)	LC/MS neg	100	40	40	100	20	40	100	40	60
3-methyl catechol sulfate (2)	LC/MS neg	60	0	20	0	0	0	40	0	20
4-methylcatechol sulfate	LC/MS neg	100	100	100	100	80	100	100	100	100
3-ethylphenylsulfate*	LC/MS neg	20	0	20	20	0	0	0	0	0
4-ethylphenylsulfate	LC/MS neg	100	100	100	100	60	60	100	80	60
4-vinylphenol sulfate	LC/MS neg	100	80	80	100	40	80	100	100	100
caffeine	LC/MS pos	80	80	100	100	100	100	100	100	100
paraxanthine	LC/MS pos	80	60	100	60	100	20	100	80	80
theobromine	LC/MS pos	80	80	100	100	100	100	100	100	100
theophylline	LC/MS neg	80	60	100	80	80	60	80	80	60
7-methylurate	LC/MS neg	100	80	100	100	100	80	100	80	80
1,3-dimethylurate	LC/MS neg	60	60	20	60	40	0	60	20	40
1,7-dimethylurate	LC/MS neg	80	60	100	80	60	40	80	80	60
1,3,7-trimethylurate	LC/MS neg	60	60	100	80	60	40	80	40	60
1-methylxanthine	LC/MS pos	60	60	80	60	60	60	80	80	60

3-methylxanthine	LC/MS pos	40	40	20	80	80	60	100	80	40
7-methylxanthine	LC/MS pos	40	40	40	80	80	80	80	80	60
5-acetylamino-6-amino-3-methyluracil	LC/MS neg	100	100	100	100	100	100	100	100	100
cotinine	LC/MS pos	20	0	20	0	0	0	0	0	0
2-piperidinone	GC/MS	60	60	40	60	80	60	100	20	60
1,6-anhydroglucose	GC/MS	100	80	100	60	80	80	100	80	100
gluconate	GC/MS	100	100	100	100	100	100	100	100	100
ergothioneine	LC/MS pos	100	100	100	100	100	100	100	100	80
erythritol	GC/MS	100	100	100	100	100	100	100	100	100
homostachydrine*	LC/MS pos	80	80	80	100	80	0	80	60	60
N-(2-furoyl)glycine	LC/MS pos	80	20	60	0	0	0	20	0	20
piperine	LC/MS pos	100	80	80	100	60	80	100	40	100
2-furoic acid	GC/MS	80	80	80	60	40	40	60	20	40
quinate	GC/MS	60	40	80	40	0	40	80	40	60
saccharin	LC/MS neg	20	0	100	40	60	100	60	100	100
stachydrine	LC/MS pos	100	100	100	100	100	100	100	80	100
thymol sulfate	LC/MS neg	100	100	60	100	80	100	100	60	40
tribuloside	LC/MS neg	0	0	0	0	20	0	0	0	0
2-hydroxyacetaminophen sulfate*	LC/MS neg	40	20	40	0	0	20	20	20	20
2-methoxyacetaminophen sulfate*	LC/MS neg	20	20	20	0	0	20	20	20	20
3-(cystein-S-yl)acetaminophen*	LC/MS pos	20	20	20	0	0	20	20	0	0
3-(N-acetyl-L-cystein-S-yl) acetaminophen*	LC/MS pos	20	0	0	0	0	0	0	0	0
4-acetaminophen sulfate	LC/MS neg	40	40	60	0	40	20	20	20	20
4-acetamidophenol	LC/MS pos	20	20	40	0	0	20	20	0	20
4-acetamidophenylglucuronide	LC/MS neg	20	20	20	0	20	20	20	20	20
ibuprofen acyl glucuronide	LC/MS neg	0	0	0	20	0	0	20	0	0
ibuprofen	LC/MS neg	0	0	0	40	0	0	20	0	0
2-hydroxyibuprofen	LC/MS pos	0	0	0	40	0	0	20	0	0
carboxyibuprofen	LC/MS neg	0	0	0	40	0	0	20	0	0
atenolol	LC/MS pos	20	20	0	0	20	20	0	0	0
desmethylnaproxen sulfate*	LC/MS neg	0	0	0	20	20	20	20	0	0
O-desmethylvenlafaxine	LC/MS pos	0	0	0	0	0	0	20	0	20
escitalopram	LC/MS pos	0	0	0	0	0	0	20	20	20
fluoxetine	LC/MS pos	0	0	0	0	0	0	20	20	20
gabapentin	LC/MS neg	20	20	20	0	0	0	0	0	0

hydrochlorothiazide	LC/MS neg	40	40	0	0	0	0	20	20	0
hydroquinone sulfate	LC/MS neg	20	20	20	0	0	0	0	0	0
hydroxypioglitazone (M-IV)	LC/MS neg	0	0	0	20	0	0	0	0	0
ketopioglitazone	LC/MS neg	0	0	0	20	0	0	0	0	0
lidocaine	LC/MS pos	0	80	20	0	80	0	0	80	0
metformin	LC/MS pos	60	40	20	100	20	20	0	0	0
metoprolol	LC/MS pos	0	0	0	0	0	0	20	20	20
metoprolol acid metabolite*	LC/MS pos	0	0	0	0	0	0	20	20	20
naproxen	LC/MS neg	40	0	0	20	20	20	40	20	0
norfluoxetine	LC/MS pos	0	0	0	0	0	0	20	20	20
omeprazole	LC/MS neg	0	0	0	0	20	20	0	0	0
pantoprazole	LC/MS neg	0	20	60	40	40	60	20	0	60
pioglitazone*	LC/MS pos	0	0	0	20	0	0	0	0	0
salicylate	LC/MS neg	40	20	40	0	20	40	20	60	40
1,2-propanediol	GC/MS	100	100	100	100	100	100	100	100	100
2-aminophenol sulfate	LC/MS neg	100	40	80	80	0	40	100	0	60
bisphenol A monosulfate	LC/MS neg	60	80	60	60	80	100	80	80	100
2-ethylhexanoate	GC/MS	100	100	100	100	100	100	100	100	100
2-hydroxyisobutyrate	GC/MS	100	100	100	100	100	100	100	100	100
dexpanthenol	LC/MS pos	0	0	0	0	0	40	0	0	0
glycerol 2-phosphate	GC/MS	100	100	100	80	80	100	100	100	100
glycolate (hydroxyacetate)	GC/MS	100	100	100	100	100	100	100	100	100

Table A3. Conserved Pre-Operation Diet (Pre-op) to Baseline (BL) Changes, $p<0.05$, $q<0.05$.				
Pathway	Biochemical Name	Fold-Change		
		SG NonT2D	SG T2D	GB T2D
Amino Acid	3-indoxyl sulfate	0.51	0.51	0.59
Lipid	oleate (18:1n9)	2.84	1.74	3.11
Lipid	cis-vaccenate (18:1n7)	2.18	1.52	2.07
Lipid	hydroxybutyrylcarnitine*	8.65	3.87	6.95
Lipid	acetoacetate	13.88	5.66	8.71
Lipid	3-hydroxybutyrate (BHBA)	18.77	5.79	11.25
Lipid	2-myristoylglycerophosphocholine	0.23	0.49	0.71
Lipid	1-linoleoylglycerophosphoethanolamine	0.51	0.52	0.6
Lipid	2-linoleoylglycerophosphoethanolamine	0.48	0.59	0.63
Lipid	1-oleoylglycerol (1-monoolein)	7.26	3.84	5.8
Lipid	1-linoleoylglycerol (1-monolinolein)	6.48	4.75	5.77
Lipid	1-linolenoylglycerol	9.97	7.16	7.06
Xenobiotics	3-methyl catechol sulfate (1)	0.15	0.21	0.02
Xenobiotics	piperine	0.15	0.09	0.36

Table A4. Conserved Post- Metabolic Surgery (Post-op) to Baseline (BL) Changes, p<0.05, q<0.05.				
Pathway	Biochemical Name	Fold-Change		
		SG NonT2D	SG T2D	GB T2D
Amino Acid	serine	2.44	4.03	2.43
Amino Acid	N-acetylserine	4.09	6.57	8.6
Amino Acid	glutamate	1.85	2.48	1.96
Amino Acid	pyroglutamine	2.17	1.71	1.34
Amino Acid	trans-urocanate	103.87	268.09	353.71
Amino Acid	cis-urocanate	76.94	329.32	543.33
Amino Acid	3-(4-hydroxyphenyl)lactate	0.65	0.66	0.65
Amino Acid	indolelactate	0.63	0.56	0.5
Amino Acid	kynurenine	0.71	0.62	0.58
Amino Acid	ornithine	6.39	17.59	23.2
Amino Acid	cysteine-glutathione disulfide	5.62	4.86	10.46
Amino Acid	5-oxoproline	4.01	7.95	8.08
Peptide	gamma-glutamylalanine	0.45	0.5	0.33
Peptide	gamma-glutamylglutamate	3.99	5.21	6.96
Peptide	gamma-glutamylthreonine	2.24	1.81	2.31
Peptide	cyclo(glu-glu)	2.79	5.63	4.98
Peptide	isoleucylvaline	0.31	0.28	0.3
Peptide	phenylalanylglutamate	4.45	10.76	4.01
Peptide	prolylglycine	4.23	3.09	7.75
Peptide	pyroglutamylvaline	81.09	111.34	180.96
Peptide	tyrosylglutamate	2.55	7.02	5.44
Peptide	valylarginine	0.25	0.4	0.36
Carbohydrate	3-phosphoglycerate	8.83	17.11	37.08
Carbohydrate	ribulose/xylulose 5-phosphate	9.26	9.94	16.01
Carbohydrate	ribulose	11.87	7.51	5.14
Carbohydrate	ribose	3.98	3.07	1.77
Carbohydrate	ribitol	0.8	2.37	0.77
Carbohydrate	xylulose	8.38	16.31	10.84
Energy	malate	2.29	4.24	3.03
Lipid	pelargonate (9:0)	0.59	0.48	0.51
Lipid	sebacate (decanedioate)	5.28	3.68	6.45
Lipid	undecanedioate	3.02	2.92	3.61
Lipid	1,11-undecanedicarboxylate	4.51	8.2	26.79
Lipid	palmitate, methyl ester	0.42	0.35	0.61
Lipid	1-myristoylglycerophosphocholine	0.27	0.31	0.28
Lipid	1-palmitoleoylglycerophosphocholine	0.37	0.39	0.4
Lipid	1-oleoylglycerophosphocholine	0.37	0.42	0.51
Lipid	1-linoleoylglycerophosphocholine	0.31	0.3	0.39
Lipid	2-linoleoylglycerophosphocholine	0.36	0.31	0.39

Lipid	1-linolenoylglycerophosphocholine	0.18	0.32	0.34
Lipid	1-dihomo-linoleoylglycerophosphocholine	0.31	0.34	0.44
Lipid	1-eicosatrienoylglycerophosphocholine	0.2	0.16	0.32
Lipid	1-arachidonoylglycerophosphocholine	0.46	0.37	0.49
Lipid	1-eicosapentaenoylglycerophosphocholine	0.15	0.2	0.5
Lipid	1-oleoylglycerophosphoethanolamine	0.45	0.68	0.56
Lipid	2-oleoylglycerophosphoethanolamine	0.46	0.7	0.64
Lipid	1-linoleoylglycerophosphoethanolamine	0.39	0.46	0.41
Lipid	2-linoleoylglycerophosphoethanolamine	0.4	0.61	0.52
Lipid	1-stearoylglycerophosphoinositol	0.29	0.38	0.57
Lipid	glycerol	3.25	8.37	3.51
Nucleotide	inosine	3.04	3.52	2.3
Nucleotide	allantoin	9.42	21.79	10.08
Nucleotide	N1-methyladenosine	0.64	0.53	0.48
Cofactors & Vitamins	nicotinamide	7.51	7.69	6.9
Cofactors & Vitamins	ascorbate (Vitamin C)	0.08	0.02	0.07
Cofactors & Vitamins	gamma-CEHC	0.38	0.24	0.17
Cofactors & Vitamins	gamma-CEHC glucuronide	0.39	0.28	0.28
Cofactors & Vitamins	heme	108.58	94.24	104.2
Cofactors & Vitamins	bilirubin (Z,Z)	0.29	0.1	0.3
Cofactors & Vitamins	bilirubin (E,E)	0.37	0.18	0.44
Xenobiotics	benzoate	0.67	0.67	0.66
Xenobiotics	catechol sulfate	0.59	0.34	0.49
Xenobiotics	piperine	0.29	0.2	0.44
Xenobiotics	saccharin	8.01	6.92	1.17

Table A5. Conserved Post- Metabolic Surgery (Post-op) to Pre-Operation Diet (Pre-op) Changes, p<0.05, q<0.05.				
Pathway	Biochemical Name	Fold-Change		
		SG NonT2D	SG T2D	GB T2D
Amino Acid	N-acetylserine	4.1	7.97	8
Amino Acid	aspartate	2.22	4.94	2.03
Amino Acid	glutamate	2.09	3.05	2.41
Amino Acid	trans-urocanate	113.05	146.41	266.35
Amino Acid	cis-urocanate	99.74	229.09	431.96
Amino Acid	3-methyl-2-oxobutyrate	0.62	0.62	0.69
Amino Acid	alpha-ketobutyrate	0.23	0.12	0.5
Amino Acid	2-hydroxybutyrate (AHB)	0.47	0.54	0.55
Amino Acid	ornithine	10.81	25.39	24.82
Amino Acid	5-oxoproline	4.98	12.04	11.32
Peptide	gamma-glutamylglutamate	3.23	6.48	7.58
Peptide	gamma-glutamylisoleucine	2.95	3.06	2.91
Peptide	gamma-glutamylthreonine	2.55	3.22	2.46
Peptide	gamma-glutamyltyrosine	1.97	2.35	2.56
Peptide	cyclo(glu-glu)	4.36	5.52	7.93
Peptide	phenylalanylglutamate	7.16	8.67	2.93
Peptide	prolylglycine	5.18	4.23	9.68
Peptide	pyroglutamylvaline	84.99	150.6	226.67
Peptide	tyrosylglutamate	2.44	5.39	4.42
Carbohydrate	3-phosphoglycerate	8.55	16.99	39.97
Carbohydrate	pyruvate	0.42	0.34	0.43
Carbohydrate	ribulose/xylulose 5-phosphate	10.7	9.94	8.37
Carbohydrate	ribulose	7.6	8.01	4.71
Carbohydrate	maltotriose	3.74	8.2	2.71
Carbohydrate	sucrose	9.85	89.85	44.52
Carbohydrate	fructose	0.38	0.78	0.35
Carbohydrate	sorbitol	11.31	8.49	7.56
Carbohydrate	mannose	0.5	0.53	0.46
Energy	malate	2.91	5.42	4.43
Lipid	linolenate [alpha or gamma]	0.3	0.47	0.53
Lipid	2-hydroxyglutarate	1.4	1.62	1.74
Lipid	undecanedioate	2.25	5.35	5.93
Lipid	1,11-undecanedicarboxylate	4.99	9.82	41.8
Lipid	acetoacetate	0.15	0.08	0.41
Lipid	1-eicosatrienoylglycerophosphocholine	0.35	0.47	0.41
Lipid	1-eicosapentaenoylglycerophosphocholine	0.24	0.4	0.45
Lipid	1-oleoylglycerol (1-monoolein)	0.28	0.25	0.13
Lipid	1-linoleoylglycerol (1-monolinolein)	0.29	0.24	0.13
Lipid	1-linolenoylglycerol	0.09	0.09	0.05

Nucleotide	hypoxanthine	4.09	7.73	2.97
Nucleotide	allantoin	21.79	57.52	32.06
Nucleotide	N1-methyladenosine	0.64	0.59	0.53
Cofactors & Vitamins	nicotinamide	11.29	4.47	5.24
Cofactors & Vitamins	ascorbate (Vitamin C)	0.05	0.05	0.06
Cofactors & Vitamins	heme	50.62	18.24	27.04
Cofactors & Vitamins	bilirubin (Z,Z)	0.4	0.14	0.3
Cofactors & Vitamins	bilirubin (E,E)	0.28	0.29	0.24
Xenobiotics	saccharin	3.48	4.19	12.65
